# Predicting the distribution of *Sphagna* (Bryophyta) in Türkiye: a perspective of present and future climate scenarios

**DOI:** 10.3389/fpls.2025.1540845

**Published:** 2025-04-08

**Authors:** Gökhan Abay, Serkan Gül

**Affiliations:** ^1^ Department of Landscape Architecture, Faculty of Engineering and Architecture, Recep Tayyip Erdogan University, Rize, Türkiye; ^2^ Department of Biology, Faculty of Arts and Sciences, Recep Tayyip Erdogan University, Rize, Türkiye

**Keywords:** ecological niche modeling, global warming, species distribution modeling, bioclimate, bryophyte, ensemble model, plant

## Abstract

Climate change is a fact that impacts all living organisms. To understand its effects, numerous methods and techniques have been refined in recent years, with species distribution modeling (SDM) being one of the most widely used. This study applied SDM to examine the distribution of seventeen *Sphagnum* species, a group of non-vascular land plants throughout Türkiye, under changing climate conditions. The study considered one global climate model (GCM)—BCC-CSM2-HR—two scenarios (SSP1-2.6 and SSP5-8.5), and two time periods (2021–2040 and 2081–2100). For the SDM analysis, a total of 211 occurrence records for whole species were used. According to the results, the future status of some species is similar to the current status, but some species show differences. Especially in the SSP5-8.5 scenario of the 2081-2100 time period, it is seen that there is a decrease in the distribution patterns of the integrals. Our study shows a simulation of the future distribution of these *Sphagnum* mosses, which have the ability to hold a lot of water, thus providing valuable information for the conservation of these species at both local and regional levels across Türkiye.

## 1 Introduction

*Sphagnum* species, also commonly referred to as peat mosses, are classified as hygrophytes. Consequently, their distribution areas are frequently associated with wetland habitats, such as peatlands (bogs and fens) and mires (Popov, 2016; Glime, 2017). Additionally, they are commonly found in oceanic wet heaths and in swamp forests (Rydin et al., 2013; Campbell et al., 2021). Among the mosses, *Sphagnum* is the most dominant in the peatlands. The dense carpet-like growth and slow rate of decay of these mosses are the two main reasons for their large volume in bogs. Additionally, the acidic pH and low concentration of dissolved solutes in bogs facilitate the growth of *Sphagnum* mosses in these habitats. The role of *Sphagnum* in peatlands is significant, with both dead and living *Sphagnum* playing an important part in carbon sequestration. Furthermore, the plants have been observed to accumulate more carbon in their bodies than is fixed by all terrestrial vegetation in a single year (Vitt et al., 2008; Gajewski et al., 2001; Zhao et al., 2023).

The number of *Sphagnum* species is 292 worldwide. The species are distributed across Europe, Asia, Africa, North and Central America, South America, Australia, New Zealand and the Pacific (Michaelis, 2019). A total of 70 *Sphagnum* taxa have been identified in all European countries (Laine et al., 2018; Hodgetts and Lockhart, 2020). The distribution of *Sphagnum* in Europe exhibits a south-to-north gradient, with the species occurring in both oceanic and continental regions. Investigations have demonstrated that northern species of *Sphagnum* are more resilient to cold autumn and winter conditions than those with a southern distribution. Furthermore, there are considerable variations in the growth response to temperature (Breeuwer et al., 2008) and drought tolerance among *Sphagnum* species (Gerdol and Vicentini, 2011; Genet et al., 2013; van de Koot et al., 2024). While climatic parameters play an important role in their distribution on a global scale (Campbell et al., 2021; Ma et al., 2022), they largely depend on soil moisture (Yoshikawa et al., 2004; Harris et al., 2005) and mineral nutrients on a local scale (Popov, 2016; Hájek and Adamec, 2009). Accordingly, Campbell et al. (2021) proposed that the distribution of *Sphagnum* species can be effectively explained by a few climatic variables that are linked to their physiological characteristics. The findings indicate that the future of *Sphagnum* diversity in Europe is most significantly influenced by alterations in water availability and seasonal temperature fluctuations.

The most recent distribution models for *Sphagnum* species have been developed on a continental scale (Oke and Hager, 2017; Campbell et al., 2021). Oke and Hager (2017) investigated the distribution of *Sphagnum* taxa in peatland areas across North America. Their findings indicated that the balance between soil moisture deficit and temperature of the driest quarter-year plays a significant role in determining the distribution of *Sphagnum* peatlands. The authors highlight that all models indicate that *Sphagnum* peatlands may expand in the future, particularly in coastal areas, under suitable climatic conditions. In a similar vein, Campbell et al. (2021) clarified the current distributions of 45 *Sphagnum* taxa in Europe, with a focus on biologically relevant climatic variables. It was emphasized that the magnitude of temperature fluctuations throughout the year represents a significant climatic factor that distinguishes current *Sphagnum* distributions. Popov (2016) correlated the distribution of six *Sphagnum* taxa from section *Sphagnum* with local climatic variables in European Russia. The results revealed that strong correlations between these species distribution and temperature and abundance of the *Sphagna* showed high correlation with maximum relative humidity in the months August and September. In a subsequent study, Popov (2018) employed a modelling approach to examine the distribution of 11 species of *Sphagnum* from section *Acutifolia*, with a focus on the gradients of climatic variables across Europea Russia and Eastern Fennoscandia. The findings indicated that high humidity is a crucial factor in promoting the abundance of *Sphagnum* species. Furthermore, the author dis-covered that the abundance of the majority of *Sphagnum* taxa is positively correlated with precipitation, humidity, and temperature in the months of August, September, and October.

A number of previous studies on the distribution modelling of *Sphagnum* species, as previously mentioned, have indicated that *Sphagnum* and peatland distributions are significantly influenced by climatic variables. To date, no study has been conducted on the distribution modelling of different *Sphagnum* species in Türkiye (formerly Turkey). The objective of this study is to examine the relationship between relevant climatic factors and *Sphagnum* distributions in Türkiye, with the aim of identifying potential future distribution areas and populations of the species.

## 2 Materials and methods

### 2.1 Samples

A review of the literature revealed the occurrence of 30 *Sphagnum* taxa in three geographical regions: Black Sea Region, Marmara Region, and Eastern Anatolia Region in Türkiye (Erata and Batan, 2020; Kırmacı and Filiz, 2021; Kürschner and Erdağ, 2021, 2023; Özen-Öztürk et al., 2023). The majority of the occurrences of *Sphagnum* taxa are in the Black Sea Region. *Sphagnum squarrosum* was a single peat moss that was exclusively found in the Ağrı province of the Eastern Anatolia region (Kürschner and Erdağ, 2021). The bryophyte locality data from the Near and Middle East (Kürschner and Erdağ, 2021) and recently published papers, including newly *Sphagnum* records for Türkiye (Erata and Batan, 2020; Kırmacı and Filiz, 2021; Kürschner and Erdağ, 2023), were used to select 17 *Sphagnum* taxa belonging to the family Sphagnaceae. The following species were identified: *Sphagnum centrale*, *S. subsecundum*, *S. platyphyllum*, *S. palustre*, *S. auriculatum*, *S. inundatum*, *S. squarrosum*, *S. compactum*, *S. girgen-sohnii*, *S. teres*, *S. fallax*, *S. capillifolium*, *S. divinum*, *S. warnstorfii*, *S. contortum*, *S. fuscum*, and *S. rubellum.* Of these, *S. centrale* was the most prevalent.

The nomenclature of *Sphagnum* species was based on the classification proposed by Hodgetts and Lockhart (2020). All taxa that were considered to be of questionable status were removed from the text. *Sphagnum aongstroemii* and *S. lescurii*, which were previously recorded from Türkiye (Çetin, 1988; Kürschner and Erdağ, 2023), were excluded from the current Turkish bryoflora (Kürschner and Erdağ, 2021) due to erroneous records. Consequently, these taxa were not subjected to further processing due to their status.

### 2.2 Brief information on the characteristics of *Sphagnums*

The gametophytic structure of *Sphagnum* taxa is characterized by a robust, erect stem, which is rarely forked. The branches are typically arranged in pendent fascicles and are densely packed at the stem tip. The number of stem leaves is less than that of branch leaves, and they are distinguished by size and shape. The sporophytes of *Sphagnum* are almost sessile. The seta is absent, and the capsule is globose, dark brown or black. They are found in acidic and poorly nutrient wetlands, mires and bogs (Kürschner and Erdağ, 2021).

### 2.3 Species occurrence data

**Figure 1:**
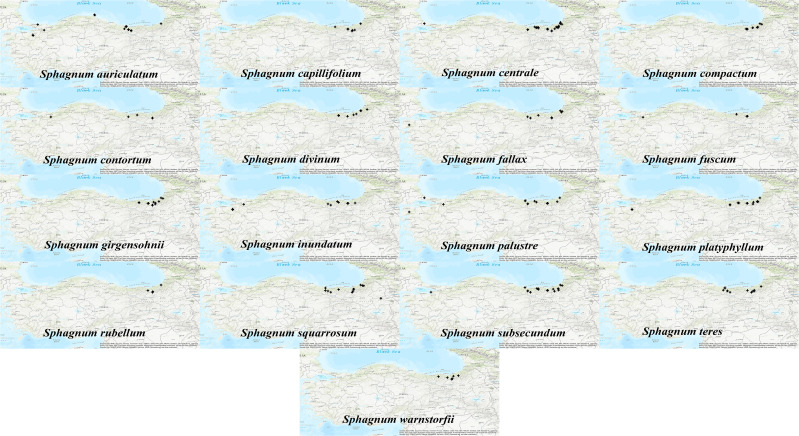
Occurrence data throughout Anatolia of *Sphagnum* mosses. The map generated with ArcMap v10.4.1 shows their distribution patterns.

A total of 235 occurrence records belonging to *Sphagnum* taxa native to Türkiye were extracted (Çetin, 1999; Özdemir and Çetin, 1999; Payne et al., 2007; Abay et al., 2009a; Abay et al., 2009b; Yayıntas, 2013; Kırmacı and Kürschner, 2013; Kırmacı and Kürschner, 2017; Abay and Keçeli, 2014; Kırmacı et al., 2016; Kırmacı et al.,2019; Kırmacı et al., 2022a; Kırmacı et al., 2022b; Özdemir and Batan, 2016; Ören et al., 2017; Söylemez et al., 2017; Erata et al., 2018; Erata et al., 2020a; Erata et al., 2020b; Erata et al.,2021a; Erata et al., 2021b; Erata et al., 2022; Canoğlu et al., 2019; Gözcü et al., 2019; Kürschner et al., 2019a; Kürschner et al., 2019b; San Keskin and Uyar, 2019; Erata and Batan, 2020; Erata and Batan, 2022; Uyar et al., 2020; Kırmacı and Filiz, 2021; Kürschner and Erdağ, 2021; Ellis et al., 2021). However, some of the *Sphagnum* species have been documented in the literature without any records of their longitude and latitude (Erdağ and Kürschner, 2021; Kürschner and Erdağ, 2021; Kırmacı et al., 2022a, 2022b). Consequently, a total of 13 *Sphagnum* taxa were excluded from further analysis due to a lack of occurrence records (fewer than five occurrences): *Sphagnum angustifolium* (C.E.O.Jensen ex Russow) C.E.O.Jensen, *S. fimbriatum* Wilson, *S. flexuosum* Dozy & Molk., *S. medium* Limpr., *S. molle* Sull., *S. subfulvum* Sjors, *S. tenellum* (Brid.) Pers. ex Brid., *S. cuspidatum* Ehrh. ex Hoffm., *S. jensenii* H.Lindb., *S. fallax* var *isoviitae* (Flatberg) Lönnell & Hassel, *S. pylaesii* Brid., *S. quinquefarium* (Braithw.) Warnst., and *S. papillosum* Lindb. Following this process, 17 *Sphagnum* species were selected for modelling, based on the meaningful predictions. Only those with a minimum of five or more longitude and latitude records (≥5 occurrences) were included, in accordance with the recommendations set out in Cerrejón et al. (2022). This resulted in a total of 211 presence records that we used in modeling, comprising 30 *S. centrale*, 20 *S. subsecundum*, 18 *S. platyphyllum*, 16 *S. palustre*, 15 *S. auriculatum*, 13 *S. inundatum* and *S. squarrosum*, 12 *S. compactum*, *S. girgensohnii*, and *S. teres*, 10 *S. fallax*, 9 *S. capillifolium* and *S. divinum*, 7 *S. warnstorfii*, 5 *S. contortum*, *S. fuscum*, and *S. rubellum* records (**Figure 1**).

### 2.4 Climatic variables

For the current variables, nineteen bioclimatic datasets were obtained from WorldClim v2.1 (accessible at https://www.worldclim.org). These datasets cover the period from 1970 to 2000, with a spatial resolution of 30 arc-seconds (~1 km²) and are available in GeoTiff (.tif) format (Fick and Hijmans, 2017). For the future variables, climate projections were downloaded from one of the global climate models (GCMs). The BCC-CSM2-HR is a high-resolution variant of the Climate System Model developed by the Beijing Climate Center (BCC). It accurately simulates the balance of global energy and effectively replicates key atmospheric patterns, including temperature, wind, precipitation, land surface air temperature, and sea surface temperature (Wu et al., 2019; İzmirli Güzel and Gül, 2023). These projections include two Shared Socio-economic Pathways (SSPs), 126 and 585, for the time periods 2021-2040 and 2081-2100, at the same 30 arc-second spatial resolution, based on CMIP6 downscaled future climate data. In the SSPs, the SSP 1-2.6 scenario envisages a significant reduction in carbon emissions by 2050, resulting in a stabilization of temperature at 1.8°C. This is considered an optimistic perspective. In contrast, the pessimistic SSP 5-8.5 scenario predicts the opposite trend. In this scenario, CO2 emissions are expected to climb until 2050, leading to an average temperature increase of 4.4°C (Pielke et al., 2022). Variance Inflation Factor (VIF) values (Marquardt, 1970) were computed using the *usdm* package (Naimi et al., 2014) and the *sdm* package (Naimi and Araújo, 2016) to reduce multicollinearity among bioclimatic variables. This process was performed separately for each species, as the method first extracts bioclimatic data from the species’ geographic locations before calculating the correlation coefficients. As a result, the variables most strongly affecting species distribution were identified for each species.

### 2.5 The execution of the model

We utilized the *sdm* package (Naimi and Araújo, 2016), which offers ensemble techniques to predict species distribution across space and time to model species distribution. This package allowed us to combine different model settings to create a consensus through ensemble models. For the ensemble modeling process, we applied five algorithms: Maxent (Maximum Entropy) (Phillips et al., 2006), GLM (Generalized Linear Models) (McCullagh and Nelder, 1989), SVM (Support Vector Machines) (Vapnik et al., 1995), Bioclim (Climate-Envelope Model) (Nix, 1986), and RF (Random Forest) (Breiman, 2001). Maxent generates species distributions based on presence and background data using bioclimatic variables (Phillips and Dudík, 2008; Phillips et al., 2024). GLM operates with presence/absence data (Carlos-Júnior et al., 2020), while SVM simulates species presence/absence (Drake et al., 2006). Bioclim requires only presence data (Grimmett et al., 2020), and RF works with presence-only data along with background samples (Valavi et al., 2021). These models, employing various methods and techniques, rank among the most effective for species distribution modeling (Naimi and Araújo, 2016). We estimated both current and future climatic predictions for each species using these ensemble models. To address the uncertainty associated with individual models, the ensemble model approach is recommended by numerous studies [Gómez et al., 2018; Hao et al., 2020; Elias et al., 2022). For ensemble modeling, we used the default parameters, splitting the data 70% for training and 30% for testing to assess model accuracy. Data partitioning was done using bootstrap, and each method was replicated 10 times.

Two statistical approaches were used to evaluate the performance of each model. The first was receiver operating characteristic (ROC) analysis, measured by the area under the curve (AUC) (Lobo et al., 2008). The AUC scale ranges from 0 to 1, where a value near 1 indicates a strong distinction between presence and pseudo-absence data, while a value of 0.5 or lower points to significant overlap between the datasets (Amaral et al., 2023). The second approach was the true skill statistic (TSS) (Allouche et al., 2006), which ranges from +1 to -1. A TSS near +1 signifies excellent model performance, while a value below 0 indicates poor performance.

## 3 Results

### 3.1 Importance of variables

For *Sphagnum auriculatum* Schimp., annual temperature range (BIO5-BIO6) (Bio7), mean temperature of the warmest quarter (Bio10), and precipitation of the wettest month (Bio13) are the most effective variables for its distribution in Anatolia, and the relative variable importance based on the correlation metric was determined to be 74.3%, 45.6%, and 20.4%, respectively (**Supplementary Figure S1**). For *S. capillifolium* (Ehrh.) Hedw., precipitation seasonality (coefficient of variation) (Bio15) and precipitation of the wettest month (Bio13) were the most important variables for the distribution of the species. The relative variable importance based on the correlation metric for these variables was also found to be 89.4% and 51.6% respectively (**Supplementary Figure S2**). For *S. centrale* C.E.O.Jensen, the maximum temperature of the warmest month (Bio5), mean diurnal range (the mean of monthly (max temp - min temp)) (Bio2) and seasonality of precipitation (coefficient of variation) (Bio15) were dominant variables with 51.1%, 25.1% and 11.5%, respectively (**Supplementary Figure S3**). Precipitation seasonality (coefficient of variation) (Bio15), precipitation of warmest quarter (Bio18) and precipitation of wettest month (Bio13) were important variables for *S. compactum* Lam. & DC based on correlation metrics 65.3%, 23.4% and 11.7%, respectively (**Supplementary Figure S4**). For *S. contortum* Schultz, precipitation of driest quarter (Bio17), precipitation of coldest quarter (Bio19) and precipitation of wettest quarter (Bio16) were the less efficient variables with relative variable importance of 78.1%, 26% and 18.9%, respectively (**Supplementary Figure S5**). Precipitation seasonality (coefficient of variation) (Bio15), with 95.1%, was the most important variable in *S. divinum* Flatberg & Hassel (**Supplementary Figure S6**). Precipitation of Wettest Month (Bio13) and Precipitation of Driest Month (Bio14) with 79.7% and 37.9%, respectively, were the variables that contributed most to the distribution of *S. fallax* (H.Klinggr.) H.Klinggr. (**Supplementary Figure S7**). However, Precipitation of warmest quarter (Bio18) with 97.7% was the most relative variable for *S. fuscum* (Schimp.) H.Klinggr. (**Supplementary Figure S8**). For *S. girgensohnii* Russow, Precipitation Seasonality (Coefficient of Variation) (Bio15), Mean Temperature of Warmest Quarter (Bio10) and Precipitation of Wettest Quarter (Bio16) were the most influential variables with 50%, 49.7% and 20.8% correlation metric, respectively (**Supplementary Figure S9**). Precipitation of the warmest quarter (Bio18), annual precipitation (Bio12), mean temperature of the coldest quarter (Bio11) and seasonality of precipitation (coefficient of variation) (Bio15) were the most effective variables with 72.6%, 60.1%, 44.6% and 10.5% respectively for the distribution of *S. inundatum* Russow throughout Anatolia (**Supplementary Figure S10**). On the contrary, for *S. palustre* L., precipitation of the wettest month (Bio13), seasonality of precipitation (coefficient of variation) (Bio15), mean temperature of the warmest quarter (Bio10) and mean temperature of the wettest quarter (Bio8) were found to be the most dominant variables with relative variable importance of 57.4%, 46.4%, 29.8% and 17.3%, respectively (**Supplementary Figure S11**). Temperature of the warmest quarter (Bio10), precipitation of the driest month (Bio14) and annual temperature range (BIO5-BIO6) (Bio7) with 83.4%, 25.9% and 18.3% were variables that can determine the distribution of *S. platyphyllum* (Lindb. ex Braithw.) Warnst. (**Supplementary Figure S12**). Nevertheless, precipitation of driest quarter (Bio17) was found to be the most effective variable for *S. rubellum* Wilson with 96% (**Supplementary Figure S13**). Mean Temperature of Warmest Quarter (Bio10), Precipitation of Wettest Month (Bio13), Precipitation Seasonality (Coefficient of Variation) (Bio15), and Mean Temperature of Driest Quarter (Bio9) were the most critical variables for *S. squarrosum* Crome (**Supplementary Figure S14**). Also, they had 62.4%, 26.1%, 21.1%, and 11.6% based on correlation metric, respectively. Precipitation seasonality (coefficient of variation) (Bio15), mean temperature of the warmest quarter (Bio10), precipitation of the wettest month (Bio13) and annual temperature range (BIO5-BIO6) (Bio7) with 39.5%, 37.2%, 28.9% and 12% correlation metric were the most prominent variables in the distribution of *S. subsecundum* Nees (**Supplementary Figure S15**). Precipitation seasonality (coefficient of variation) (Bio15), precipitation of the wettest quarter (Bio16) and mean temperature of the warmest quarter (Bio10) showed the influence on the distribution of *S. teres* (Schimp.) Ångstr. based on correlation metrics of 46%, 44.7% and 26.7% respectively (**Supplementary Figure S16**). Precipitation of warmest quarter (Bio18), precipitation of wettest quarter (Bio16) and precipitation of coldest quarter (Bio19) with 62.5%, 62.1% and 41.7% respectively were found to be important for *S. warnstorfii* Russow (**Supplementary Figure S17**).

### 3.2 The performance of models

The evaluation of the ensemble model was conducted using both threshold-dependent (TSS) and threshold-independent (AUC) statistics. For the species *Sphagnum auriculatum*, *S. capillifolium*, *S. centrale*, *S. compactum*, *S. fallax*, *S. fuscum*, *S. girgensohnii*, *S. inundatum*, *S. palustre*, *S. platyphyllum*, *S. squarrosum*, *S. teres*, and *S. warnstorfii*, the Maxent model showed the best performance among five implemented models, with AUC scores of 0.96, 0.97, 0.97, 0.96, 0.93, 0.95, 0.98, 0.98, 0.95, 0.98, 0.96, 0.98, 0.98, and corresponding TSS values of 0.89, 0.96, 0.91, 0.94, 0.90, 0.92, 0.96, 0.95, 0.90, 0.95, 0.91, 0.94, and 0.98 (**Table 1**). In contrast, for *S. contortum*, the best-performing model was SVM, with an AUC of 0.99 and a TSS of 0.98. Additionally, both GLM and Maxent performed similarly for *S. divinum* and *S. subsecundum*, with AUC values of 0.96 and 0.97 and TSS values of 0.92 and 0.90. However, for *S. rubellum*, the highest TSS value was 0.95, while both Maxent and GLM had the same AUC score of 0.96 (**Table 1**).

**Table 1:** Model performance for *Sphagnum* species.

Methods	*Sphagnum auriculatum*		*Sphagnum capillifolium*		*Sphagnum centrale*		*Sphagnum compactum*		*Sphagnum contortum*	
	AUC	TSS	AUC	TSS	AUC	TSS	AUC	TSS	AUC	TSS
GLM	0.93	0.83	0.94	0.93	0.97	0.9	0.95	0.92	0.8	0.76
RF	0.93	0.83	0.97	0.95	0.97	0.86	0.91	0.87	0.92	0.87
MAXENT	0.96	0.89	0.97	0.96	0.97	0.91	0.96	0.94	0.95	0.93
BIOCLIM	0.68	0.38	0.84	0.69	0.73	0.46	0.8	0.62	0.77	0.54
SVM	0.83	0.7	0.84	0.82	0.81	0.72	0.79	0.75	0.99	0.98
Methods	*Sphagnum divinum*		*Sphagnum fallax*		*Sphagnum fuscum*		*Sphagnum girgensohnii*		*Sphagnum inundatum*	
	AUC	TSS	AUC	TSS	AUC	TSS	AUC	TSS	AUC	TSS
GLM	0.96	0.92	0.86	0.79	0.89	0.81	0.97	0.94	0.89	0.86
RF	0.95	0.86	0.93	0.85	0.89	0.84	0.96	0.9	0.96	0.9
MAXENT	0.96	0.92	0.93	0.9	0.95	0.92	0.98	0.96	0.98	0.95
BIOCLIM	0.84	0.7	0.76	0.57	–	–	0.73	0.46	0.83	0.66
SVM	0.94	0.9	0.94	0.89	0.93	0.92	0.66	0.58	0.94	0.86
Methods	*Sphagnum palustre*		*Sphagnum platyphyllum*		*Sphagnum rubellum*		*Sphagnum squarrosum*		*Sphagnum subsecundum*	
	AUC	TSS	AUC	TSS	AUC	TSS	AUC	TSS	AUC	TSS
GLM	0.93	0.85	0.97	0.94	0.96	0.95	0.93	0.86	0.95	0.86
RF	0.9	0.81	0.97	0.89	0.91	0.88	0.92	0.82	0.97	0.9
MAXENT	0.95	0.9	0.98	0.95	0.96	0.94	0.96	0.91	0.97	0.9
BIOCLIM	0.79	0.61	0.81	0.62	0.74	0.53	0.68	0.37	0.71	0.44
SVM	0.91	0.85	0.89	0.81	0.85	0.84	0.88	0.82	0.9	0.83
Methods	*Sphagnum teres*		*Sphagnum warnstorfii*							
	AUC	TSS	AUC	TSS						
GLM	0.97	0.93	0.95	0.94						
RF	0.96	0.91	0.95	0.93						
MAXENT	0.98	0.94	0.98	0.98						
BIOCLIM	0.8	0.61	0.75	0.5						
SVM	0.96	0.89	0.94	0.93						

### 3.3 Present and future distribution patterns of *Sphagnum* species in Türkiye

**Figure 2:**
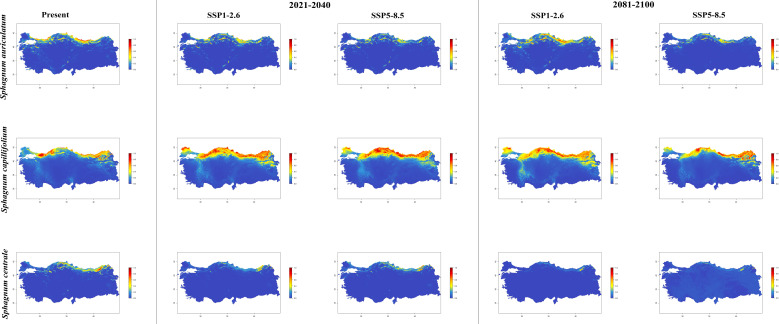
The distribution patterns of *Sphagnum auriculatum*, *Sphagnum capillifolium*, and *Sphagnum centrale* under present and future conditions based on consensus ensemble modelwfi .

**Figure 3:**
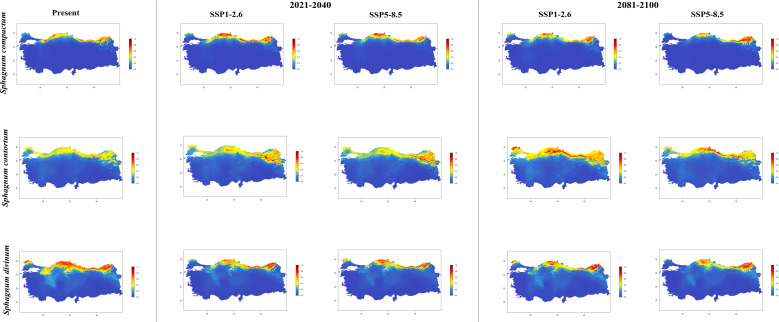
The distribution patterns of *Sphagnum compactum*, *Sphagnum contortum*, and *Sphagnum divinum* under both present and future climatic conditions based on consensus ensemble model.

**Figure 4:**
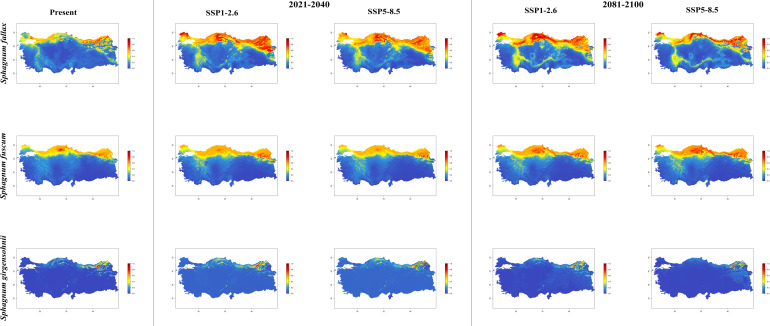
The distribution patterns of *Sphagnum fallax*, *Sphagnum fuscum*, and *Sphagnum girgensohnii* under both present and future climatic conditions based on consensus ensemble model.

**Figure 5:**
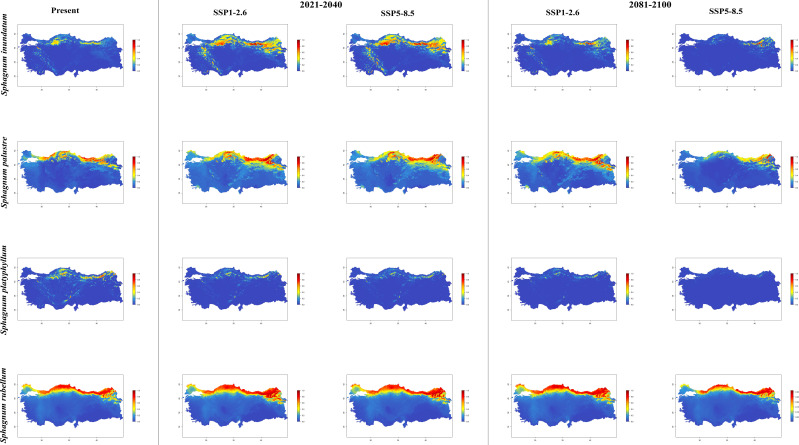
The distribution patterns of *Sphagnum inundatum*, *Sphagnum palustre*, *Sphagnum platyphyllum*, and *Sphagnum rubellum* under both present and future climatic conditions based on consensus ensemble model.

**Figure 6:**
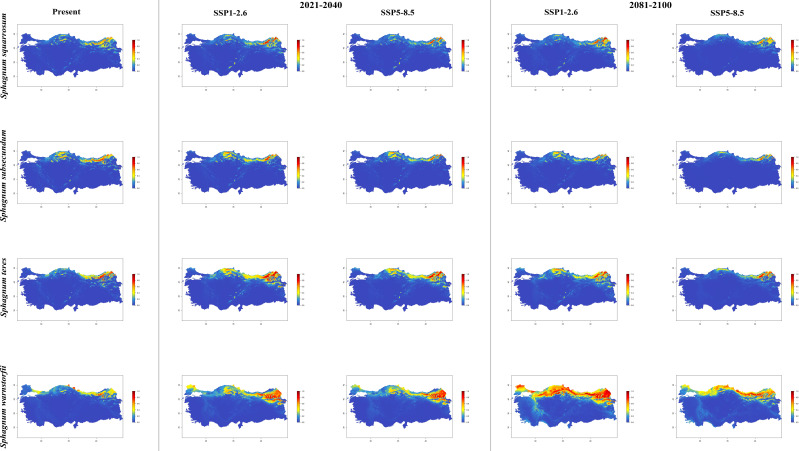
The distribution patterns of *Sphagnum squarrosum*, *Sphagnum subsecundum*, *Sphagnum teres*, and *Sphagnum warnstorfii* under both present and future climatic conditions based on consensus ensemble model.

In general, the majority of *Sphagnum* species are anticipated to alter their current distribution in accordance with the prediction models employed. Some species have decreased in suitable habitats, while others have increased. While the suitable habitats for *Sphagnum auriculatum* are in the western part and central part of the Black Sea Region, this suitability will decrease in both scenarios of the 2021-2040 time period, and even in the SSP5-8.5 scenario of the 2081–2100-time interval, a similar pattern emerges in the SSP1-2.6 scenario, although the situation is similar to the current distribution in the SSP1-2.6 scenario (**Figure 2**). In *S. capillifolium*, suitability increased in all time intervals and scenarios com-pared to the present (**Figure 2**). However, in *S. centrale*, suitability decreased across all time intervals and scenarios (**Figure 2**). For *S. contortum*, suitability will increase in the future compared to the present, and this will be slight for *S. compactum* (**Figure 3**). For *S. divinum*, however, partial decreases in suitability are predicted for all periods and scenarios (**Figure 3**). *S. fallax* is already very different, with increased fitness in all time intervals and scenarios (**Figure 4**). On the contrary, neither *S. fuscum* nor *S. girgensohnii* shows this pattern, while all future predictions are similar to the present (**Figure 4**). A similar pattern also occurs in *S. rubellum* (**Figure 5**). The present suitable habitats of *S. inundatum* are the interior parts of the Black Sea Region. It is predicted that the suitability increases in both scenarios of the 2021–2040-time interval, and this situation partially continues in the SSP1-2.6 scenario of the 2081–2100-time interval, but changes in SSP5-8.5 (**Figure 5**). A similar pattern is observed in *S. palustre* (**Figure 5**). On the contrary, the distribution pattern of *S. platyphyllum* shows a decrease in conformity and even a complete loss of conformity in the SSP5-8.5 scenario of the 2081–2100-time interval (**Figure 5**). The current suitability for *S. squarrosum*, S*. subsecundum*, and *S. teres* species is mainly in the eastern part of the Black Sea Region. Slight changes are seen in the future, with the greatest decrease in all of them in the SSP5-8.5 scenario for the time interval 2081-2100 (**Figure 6**). However, the current distribution pattern of *S. warnstorfii* is very different from the future, with an increase in suitability in all time intervals and scenarios, with the highest increase in the 2081–2100-time interval in the SSP1-2.6 scenario (**Figure 6**).

## 4 Discussion

National studies show that *Sphagnum* species are established in the northern parts of Türkiye, especially near the eastern part of the Black Sea Region. Apart from this, *Sphagnum* taxa are also present in the western part of the Black Sea Region and Marmara Region (Kürschner and Erdağ, 2021; Kırmacı et al., 2022a). Maps shows a simulation of the future distributions for the *Sphagna* based on current geographical distribution records and climatic data (**Figures 2**-**6**).

In the period between 2021 and 2040, the distribution of *Sphagnum capillifolium* is expected to expand in the region between 40°N and 42°N. There is an increase in the distribution areas of *S. capillifolium* in the periods 2021-2040 and 2081-2100 according to the future climatic scenarios. Popov (2018) reported a positive correlation between *S. capillifolium* and high humidity, as well as a negative correlation between the species and both monthly and annual mean temperatures in the Eastern European Plain and Eastern Fennoscandia. Additionally, the species was observed to flourish in regions with an annual precipitation exceeding 450–460 mm and an average annual temperature below +7°C. However, the results of our study indicate that the distribution areas of the species are undergoing a positive development in response to the predicted temperature increase for the coming years, as illustrated in the simulated maps. The selected climate scenarios (SSP1-2.6 and SSP5-8.5) and the applied model indicate that there will be reductions in the current distribution areas for *S. auriculatum* between 2021 and 2040, with a further decline in the 2081-2100 period. Indeed, the data associated with the SSP5-8.5 scenario points to a more pessimistic outlook with regard to habitat loss but does not disappear at all. In accordance with the projected climate scenarios for the period between 2081 and 2100, it can be stated that *S. centrale* will experience a notable reduction in its distribution areas as a consequence of rising temperatures, with the potential for even greater habitat loss. Popov (2016) indicates that the abundance of *S. centrale* exhibits a markedly positive correlation with precipitation and relative humidity during the period from late summer to mid-autumn (August-October), and a negative correlation with temperature during this same period. Furthermore, the author indicates that the presence of this species is inversely correlated with summer temperatures. The fact that the species has lost suitable habitats in its current local distribution in the eastern part of the Black Sea Region with the increase in temperature under different climate scenarios in the coming years supports what was described by Popov (2016).

*Sphagnum compactum* (shown in the period 2021-2040 and the scenario SSP1-2.6) and *S. contortum* (shown in the period 2081-2100 and the scenario SSP1-2.6) reaches the highest frequency in the northern distributions of Türkiye with the simulated suitable habitats. Two distinct scenarios for the forthcoming two decades (2021-2040) revealed an eastward expansion in the distribution of *S. contortum*, accompanied by a southward expansion of the species’ distribution area between the latitudes 38°N and 42°N. In their 2007 study, Hájková and Hájek (2007) reported that *S. contortum* is a spring-growing species that typically occupies streams. They noted that it is relatively common in Bulgarian mires. However, this species is categorized as VU, EN and LC in the Red List for many different countries (Papp et al., 2010; Ştefănuţ and Goia, 2012; Sérgio et al., 2007; Lazarević et al., 2016; Mišíková et al., 2020). The findings of our study indicate that the species is likely to expand its range in accordance with projected future climate scenarios. The distribution maps simulated for future years indicate a decrease in the distribution of *S. divinum*, with a particularly notable decline observed in the western and central parts of the Black Sea Region. According to both climate scenarios, the habitat loss in this area is predicted to occur between 40°N and 42°N between the years 2081 and 2100. According to Kırmacı et al. (2022b), *S. divinum* was evaluated as Near Threatened within the IUCN categories. According to the findings of our study, the fact that the species shows habitat loss under future climate scenarios supports the idea that the species will become threatened in the near future as stated by the authors.

It is projected that the distribution of *Sphagnum fallax* will expand significantly in comparison to its current range under the selected climate scenarios for the forthcoming years. Furthermore, there are indications of an expansion from the south-eastern part of the Marmara Region to the western part of the Mediterranean Region. The results presented herewith diverge from those observed in the studies conducted by Bragazza et al. (2016); Norby et al. (2019), and Jassey and Signarbieux (2019). Bragazza et al. (2016) presents the findings of a transplantation experiment involving peat mesocosms, which were relocated from high to low altitude in order to simulate a mean annual temperature approximately 5°C higher and a mean annual precipitation approximately 60% lower over a three-year period. The authors posited that the decline in annual productivity observed in the peat moss *S. fallax* in transplanted mesocosms was attributable to a combination of physical and biological constraints, namely water scarcity and light competition, respectively. Furthermore, it was demonstrated that the productivity of *S. fallax* declined by 60% in mesocosms that had been relocated to a warmer (+5°C) environment. In a further study, Norby et al. (2019) investigated the impact of experimental warming on the decline of *S. fallax* in a bog environment. Their hypothesis was based on the observation that *S. fallax* exhibited a 50% reduction in occurrence when transplanted to a location with a higher temperature (+5°C) and lower humidity. Regarding these contrasting results, Norby et al. (2019) argued that mound-pit microtopography has a greater influence on *Sphagnum* responses to warming than species-specific traits. Jassey and Signarbieux (2019) demonstrated that the negative effects of summer droughts on *S. fallax* water content were exacerbated by warming, resulting in an even sharper decrease in water content. The findings of our study suggest that the projected increase in temperature over the coming decades will not have a detrimental impact on the distribution of *S. fallax*. The estimated distribution maps indicate a slight decrease in the distribution of *S. fuscum* in the western part of the Black Sea Region and a slight increase in the north-eastern part of Türkiye over the next 20 years. However, the SSP1-2.6 and SSP5-8.5 scenarios predict a significant increase in the current distribution areas of the same species between 2081 and 2100. The distribution of *S. fuscum* under the SSP1-2.6 and SSP5-8.5 scenarios within the 2081-2100 period is consistent with the findings of different studies. Breeuwer et al. (2008) conducted a greenhouse experiment to investigate the impact of varying temperature treatments. The results demonstrated that the lowest to the highest temperature treatments in monocultures led to an increase in biomass production of *S. fuscum*. Naumov and Kosykh (2011) observed that the species possesses the capacity to retain moisture for extended periods, which may be regarded as an adaptation to desiccation. Furthermore, Bengtsson et al. (2021) demonstrated that *S. fuscum* exhibits a diminished reliance on a sustained wet climate, and the moss display enhanced stability and resilience to climatic fluctuations. It is capable of maintaining photosynthetic activity during periods of drought and in the absence of precipitation. The species occurrences in the aforementioned period and scenarios appear to corroborate the hypothesis put forth by the authors in relation to global warming. The most significant habitat loss for *S. girgensohnii* was observed in the SSP5-8.5 climate scenario between 2081-2100.

The habitat and abundance of *Sphagnum inundatum* in the Black Sea region during the period 2021-2040, according to both the SSP1-2.6 and SSP5-8.5 scenarios. While there is a notable expansion in the current distribution of *S. palustre* between 2021 and 2040, it is projected that this species will experience a considerable reduction in its habitat under the SSP5-8.5 climate scenario during the 2081-2100 period. It is likely that *S. palustre* will persist in a restricted area, particularly in the northeastern regions of the country. According to the results of the regression analysis in the study of Popov (2016), the climatic factors affect the occurrence of *S. palustre* in the East European Plain. Also, the author explained that the distribution of the species with the increasing of summer temperatures. This idea supports the species distributions in the 2021-2040 and 2081-2100 periods and targeted to the climate scenarios, except the scenario SSP5-8.5 in the year 2081-2100. The future of *S. plathyphyllum* appears to be rather pessimistic. It is assumed that the species will experience a contraction in its habitat within the next twenty years. Furthermore, it is predicted that it will become extinct in all areas of Anatolia throughout the 2081-2100 period, most notably under the SSP5-8.5 climate scenario. *S. plathyphyllum* is typically found in wet lo-cations, where it grows submerged or in close proximity to the water surface (Daniels and Eddy, 1990). Similarly, the results of the study conducted by Campbell et al. (2021) indicate that the future of *Sphagnum* diversity in Europe is most strongly contingent upon alterations in water availability and seasonal temperature fluctuations. The distribution map of *S. plathyphyllum*, which was created as a result of the model applied by taking into account the 4.4°C temperature increase predicted between 2081-2100, illustrates the impact of habitat loss. This can be explained by the reduction of water availability and the restriction in the potential distribution of the species due to a drier climate, which is a probable consequence of climate change in the eastern part of the Black Sea Region. While the long-term survival of the species *S. rubellum* is not at risk, it is expected that the species will continue to expand its distribution range towards the northern regions of Anatolia. In a study conducted by Robroek et al. (2007), it was observed that the biomass production of *S. rubellum* was significantly higher at elevated temperatures than at lower temperatures. According to the authors, this finding aligns with the observation that this species has a more southern distribution. Our findings support the idea of the mentioned study, in connection with global warming. Ma et al. (2022) indicated that the precipitation of the driest month is one of the most significant environmental variables for *S. rubellum*, with 52.7% of the species currently occurring under such conditions. Similarly, Oke and Hager (2017) found that the temperature of driest quarter as second variable for the occurrence of *S. rubellum* on peatlands in North America using single-and multi-species models with 26.9%. This finding is consistent with the precipitation of the driest quarter result obtained for the moss, which demonstrated a value of 96%. Therefore, the results indicate that extreme dry periods do not impact the distribution of *S. rubellum*, as illustrated in the simulated maps.

In the long term and under the SSP5-8.5 climate scenario, the greatest habitat loss and abundance decline will be experienced by *Sphagnum subsecundum* and *S. squarrosum*, respectively, in comparison to their current habitats. Daniels and Eddy (1990) reported that the *S. subsecundum* is most commonly found in habitats adjacent to streams or on wet and peaty slopes. In light of the aforementioned ecological preferences of the species, it is anticipated that there will be a partial reduction in the extent of the species’ habitat as a consequence of rising temperatures in the coming years. The applied model indicates that new distribution areas and an increase in existing habitats for *S. teres* towards the western part of the Black Sea Region are likely to occur between 2021 and 2040, according to both climate scenarios. However, it suggests that the species may persist only in North-East Anatolia during the 2081-2100-time period and under the SSP5-8.5 climate scenario. Despite the anticipated increase in temperature and aridity over the 2081-2100 period, the most favorable outlook was identified for *S. warnstorfii*. The most noteworthy observation was made in the 2081-2100-time period and under the SSP1-2.6 climate scenario. The model indicates that the species will experience significant distribution in Thrace, the western and central parts of the Black Sea Region, the north-east of the country and Eastern Anatolia Region during the specified time period and climate scenario. Pakarinen (1979) proposed that this extensive distribution may be attributable to the existence of genetically differentiated ecotypes within *S. warnstorfii*. Mikulášková et al. (2015) concluded that genetics may explain the relatively broad niche of *S. warnstorfii*, which consists of numerous cryptic species with broadly overlapping geo-graphical ranges (Hájková and Hájek, 2007). Flatberg et al. (2006) observed the formation of hybrids between the calcium-tolerant *S. warnstorfii*. Brauer et al. (2023) postulated that natural hybridization may serve to mitigate the vulnerability of species to climate change. The results of our study generally corroborate the aforementioned observations. The projected expansion of this species in response to a temperature increase could be attributed to genetically determined ecotypic differentiation.

Variables that contribute the most to the model applied for the distribution of all *Sphagnum* species were precipitation seasonality, and mean temperature of the warmest quarter, respectively. The precipitation of the wettest month, the precipitation of the wettest quarter, and the precipitation of the warmest quarter were identified as the other bioclimatic variables that were found to be significant in the creation of species distribution maps, among the remaining variables. The most relative variable can determine the distribution of *Sphagnum fuscum* was noticed as the precipitation of warmest quarter (Bio18) with 97.7%. Unlike *S. fuscum*, the precipitation of the driest quarter (Bio17) was identified as the most effective variable for *S. rubellum*, with a correlation coefficient of 0.96. Seasonality precipitation (Bio15) was the least effective variable in determining the distribution of *S. inundatum* in Anatolia with a value of 10.5%. Cong et al. (2020) asserts that *Sphagnum* development is significantly influenced by climatic factors, and that optimal conditions for *Sphagnum* growth are characterized by specific humidity and temperature levels. Bengtsson et al. (2021) stated that temperature is a significant climatic factor influencing plant production, with increased precipitation expected to enhance growth. Also, Ma et al. (2022) reports that the mean temperature of the coldest quarter and precipitation of the driest month are the primary factors influencing the habitat availability of *Sphagnum* mosses. Similar to the studies mentioned above, the results of our study confirm these hypotheses.

## 5 Conclusions

The objective of this study is to predict the pattern of change in the national-scale distribution of *Sphagnum* mosses in Türkiye under future climate change scenarios. Given the differing rates at which *Sphagnum* species respond to future climate scenarios, the ranges of species in Türkiye are subject to constant flux. Climate scenarios for Türkiye predict a drastic reduction in the distributions of *S. auriculatum*, *S. centrale* and *S. plathyphyllum* by the end of the twenty-first century. In light of the potential climatic variables that could negatively impact the distribution of all three species, temperature was identified as the most crucial parameter. The total disappearance of the three *Sphagnum* species from their native habitats is undoubtedly associated with a consistent decline in precipitation levels, which has led to a notable reduction in the local water balance. The rise in temperature had no adverse impact on the habitats where *S. rubellum* was present, whereas it had a beneficial effect on *S. capillifolium*, *S. contortum*, *S. fallax*, *S. fuscum*, and *S. warnstorfii*. It is predicted that species that will benefit from climate change will be those that are able to survive in habitats that are not at risk of being adversely affected by a changing climate, and which have the capacity to disperse and colonize new areas. It can be reasonably inferred that certain characteristics of *Sphagnum* may render them less susceptible to fluctuations in temperature. This study is of significant importance for the conservation of *Sphagnum* species with specialized habitats, as well as for future research on the responses of these species to climate change.

Future research should include an assessment of the potential impacts of climate change on *Sphagnum* species in Türkiye and conservation strategies to mitigate negative impacts. The aim should be to develop conservation strategies to protect *Sphagnum* species and associated habitats in Türkiye based on available ecological data and field surveys. Changes in *Sphagnum* habitats, including changes in hydrology and soil composition, should be analyzed when assessing ecological impacts. In assessing conservation and habitat management strategies, priority conservation areas for *Sphagnum* species should be identified, *ex situ* and *in situ* conservation measures should be developed, including habitat restoration and climate adaptation techniques, and stakeholders (e.g. policy makers, conservation organizations) should be involved to integrate the findings into national conservation plans. The expected outcomes are a detailed projection of the future distribution of *Sphagnum* species, insights into the wider ecological consequences of possible climate change on *Sphagnum* habitats in Türkiye, and feasible conservation recommendations to mitigate habitat loss and biodiversity decline.

In summary, this study provides important, data-driven insights to help inform local and regional conservation strategies, support climate adaptation efforts, and guide water and ecosystem management policies. The findings contribute directly to achieving key global goals for climate action, water conservation and biodiversity conservation.

## Data Availability

The raw data supporting the conclusions of this article will be made available by the authors, without undue reservation.
