# Supplementary material for: Predicting the distribution of Sphagna (Bryophyta) in Türkiye: a perspective of present and future climate scenarios
Source: Front Plant Sci. 2025 Apr 8;16:1540845. doi: 10.3389/fpls.2025.1540845 (PMC12011740; doi:10.3389/fpls.2025.1540845)
Supplement: Supplementary file 1 [file Table1.docx]

**Supplementary Materials**

**Predicting the distribution of Sphagna (Bryophyta) in Türkiye: a perspective of present and future climate scenarios**

Gökhan Abay^1^, Serkan Gül^2*^

^1^Department of Landscape Architecture, Faculty of Engineering and Architecture, Recep Tayyip Erdogan University, Rize, 53100, Türkiye

^2^Department of Biology, Faculty of Arts and Sciences, Recep Tayyip Erdogan University, Rize, 53100, Türkiye

*corresponding author: serkan.gul@erdogan.edu.tr


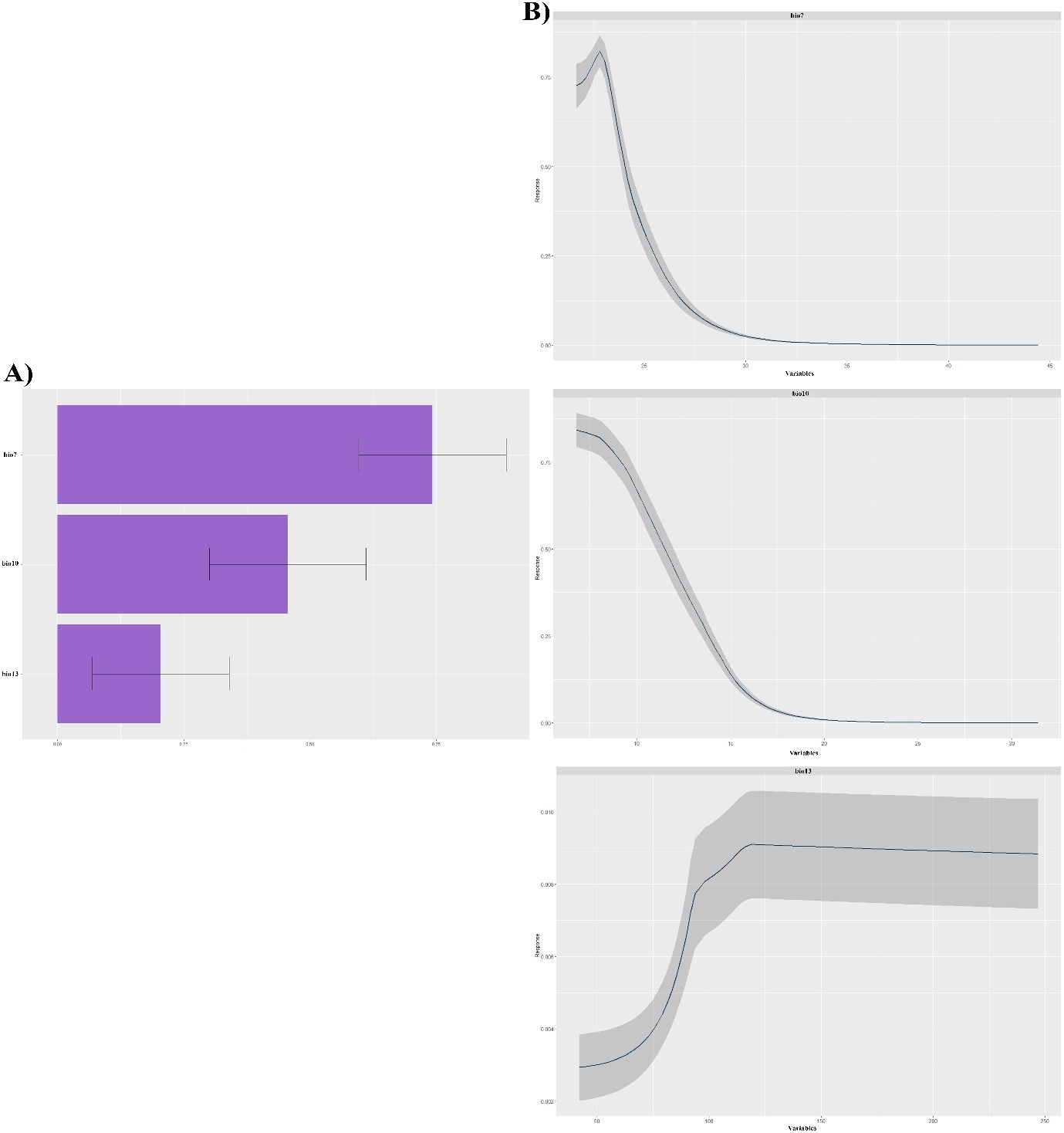


**Figure S1.** A) It shows relative variable importance for *Sphagnum auriculatum*, B) This represents the response curve of the most important variables to model predicting *Sphagnum auriculatum*.


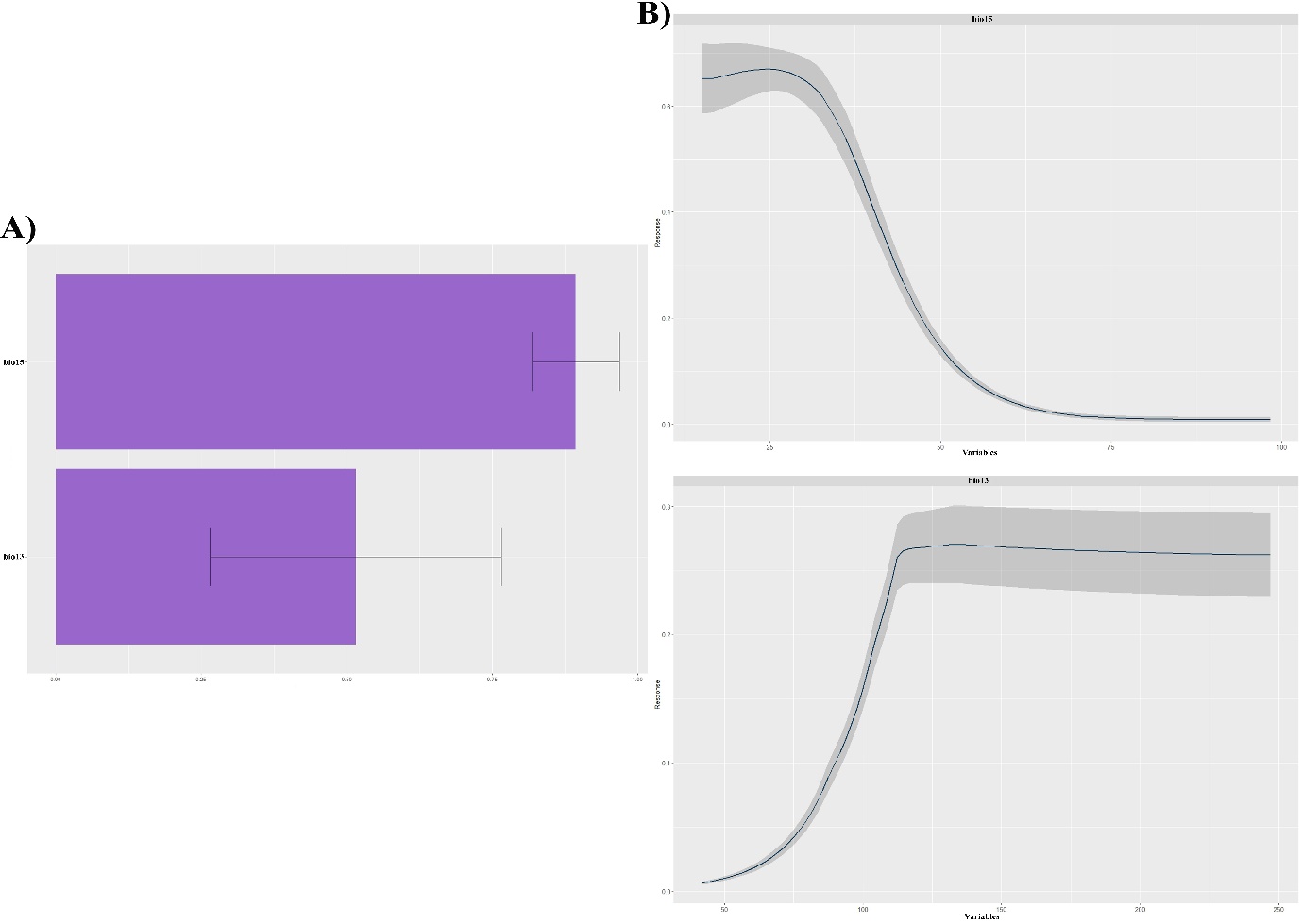


**Figure S2.** A) It shows relative variable importance for *Sphagnum capillifolium*, B) This represents the response curve of the most important variables to model predicting *Sphagnum capillifolium*


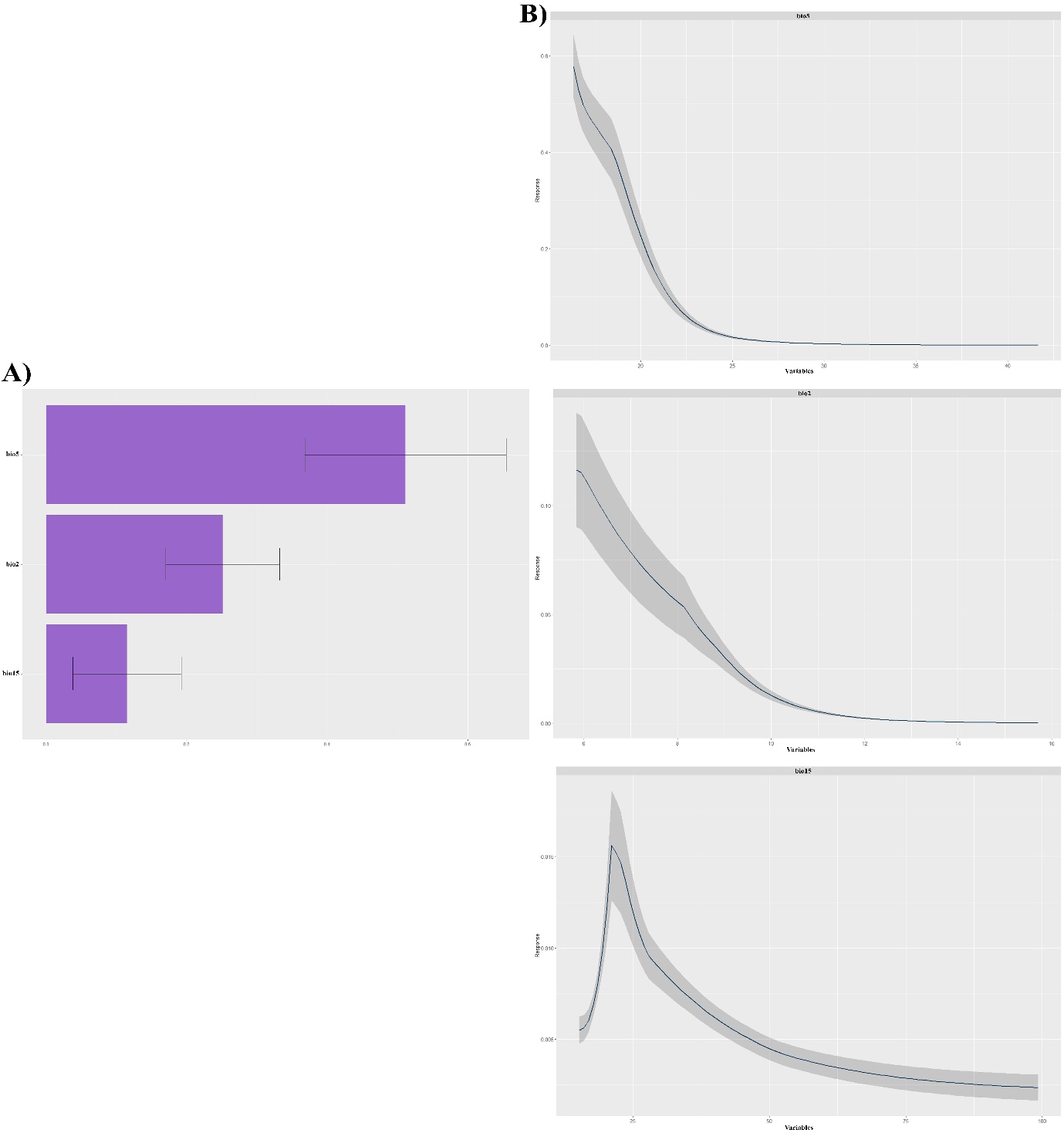


**Figure S3.** A) It shows relative variable importance for *Sphagnum centrale*, B) This represents the response curve of the most important variables to model predicting *Sphagnum centrale*.


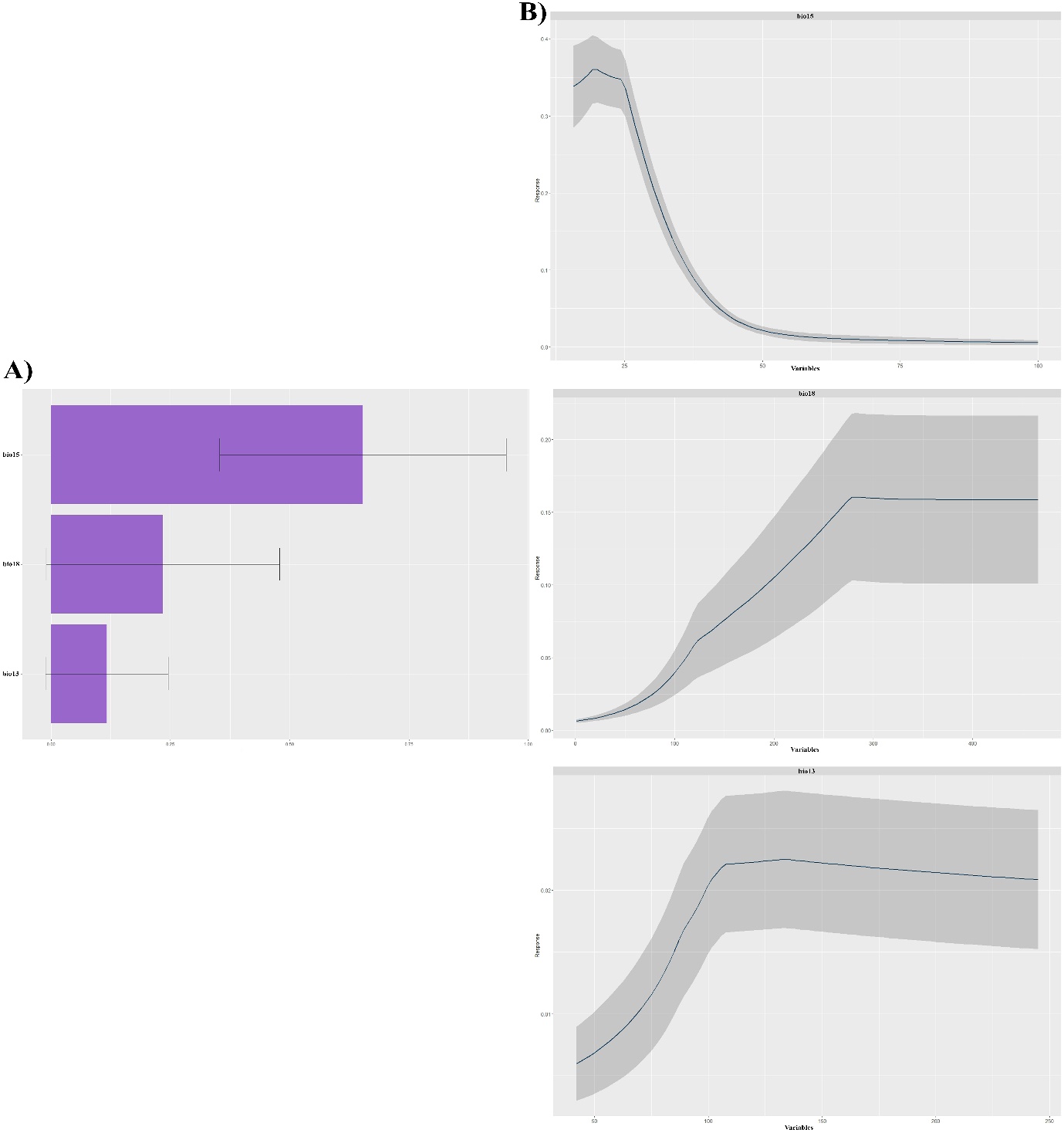


**Figure S4.** A) It shows relative variable importance for *Sphagnum compactum*, B) This represents the response curve of the most important variables to model predicting *Sphagnum compactum*.


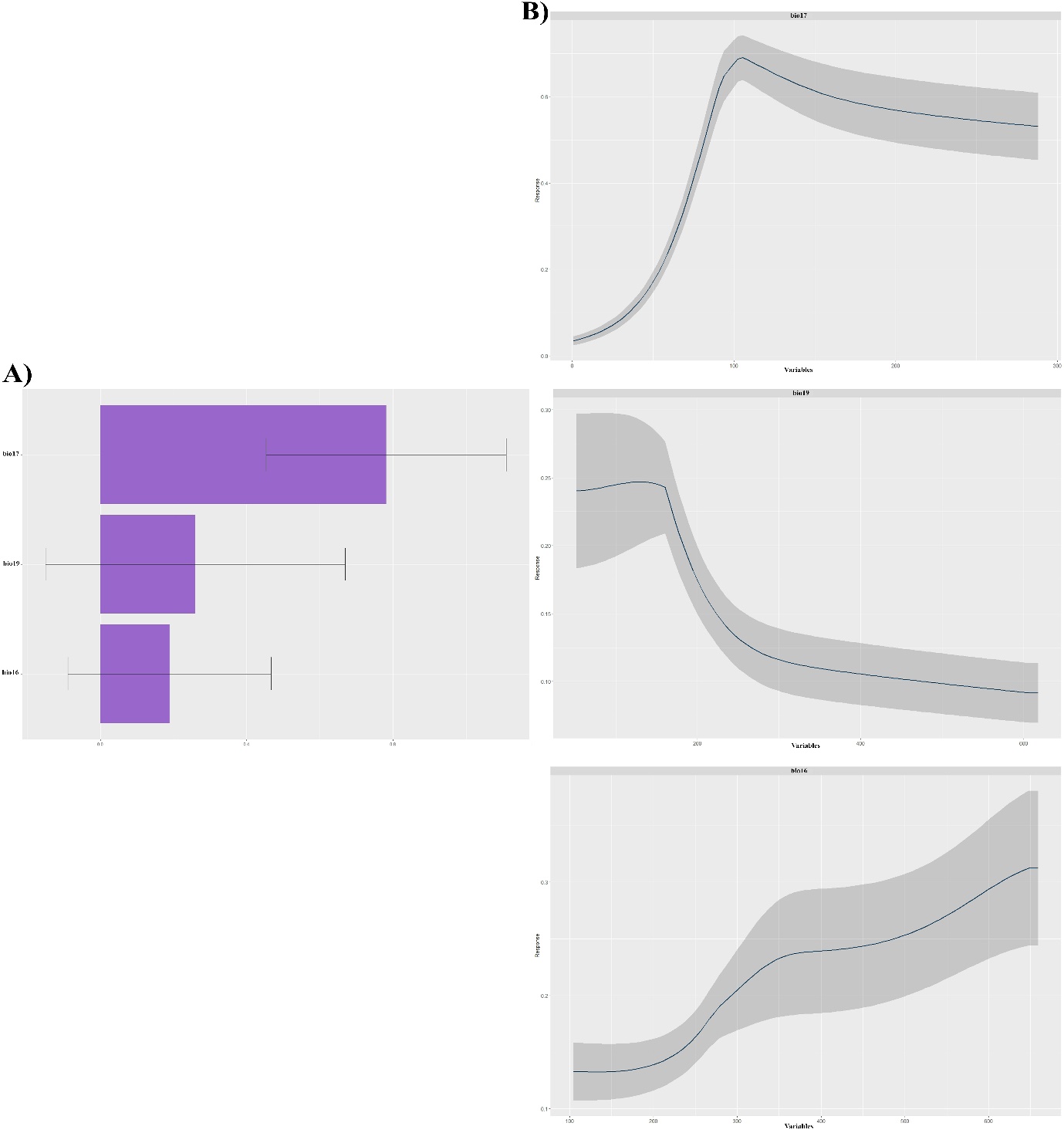


**Figure S5.** A) It shows relative variable importance for *Sphagnum contortum*, B) This represents the response curve of the most important variables to model predicting *Sphagnum contortum*.


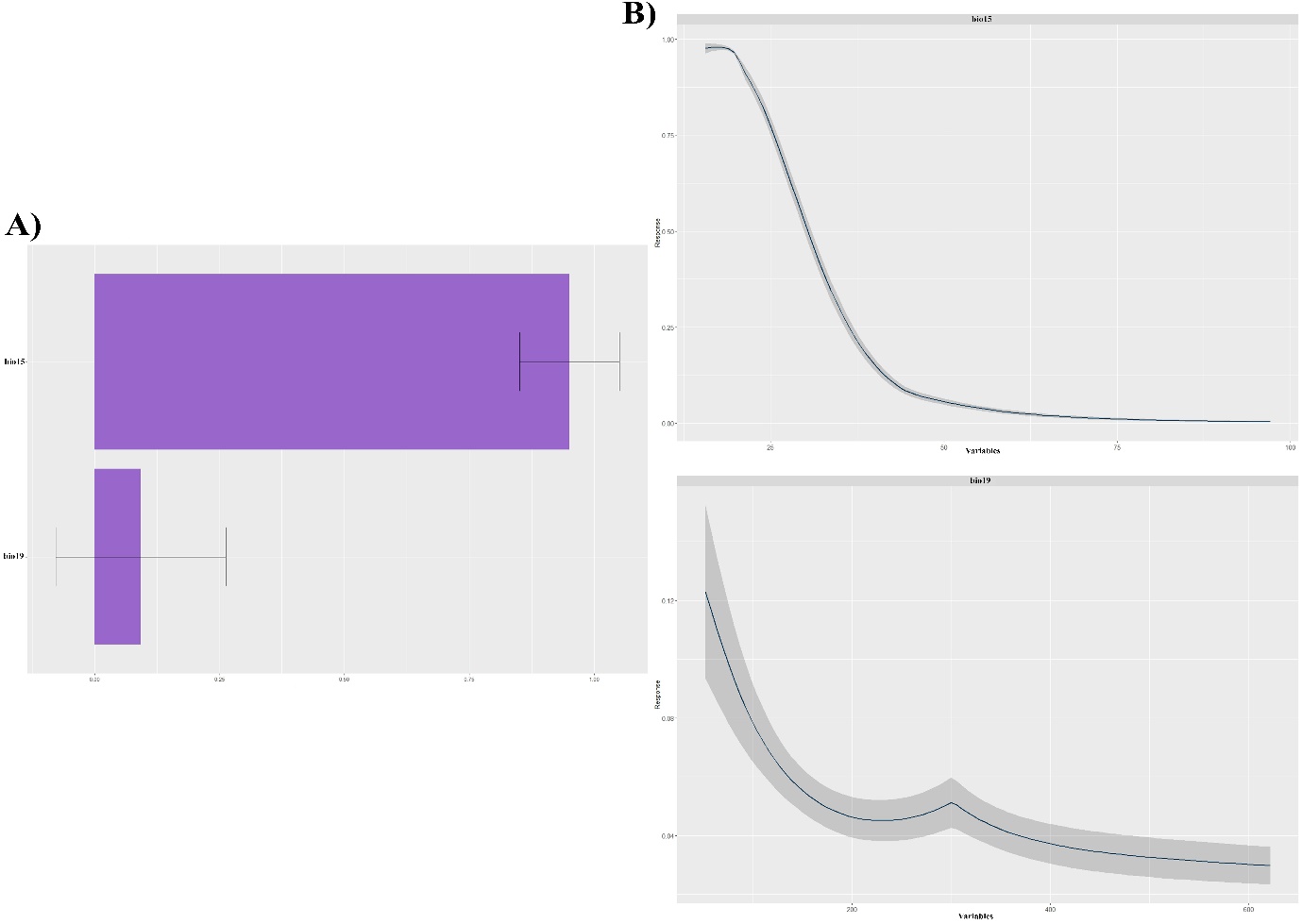


**Figure S6.** A) It shows relative variable importance for *Sphagnum divinum*, B) This represents the response curve of the most important variables to model predicting *Sphagnum divinum*.


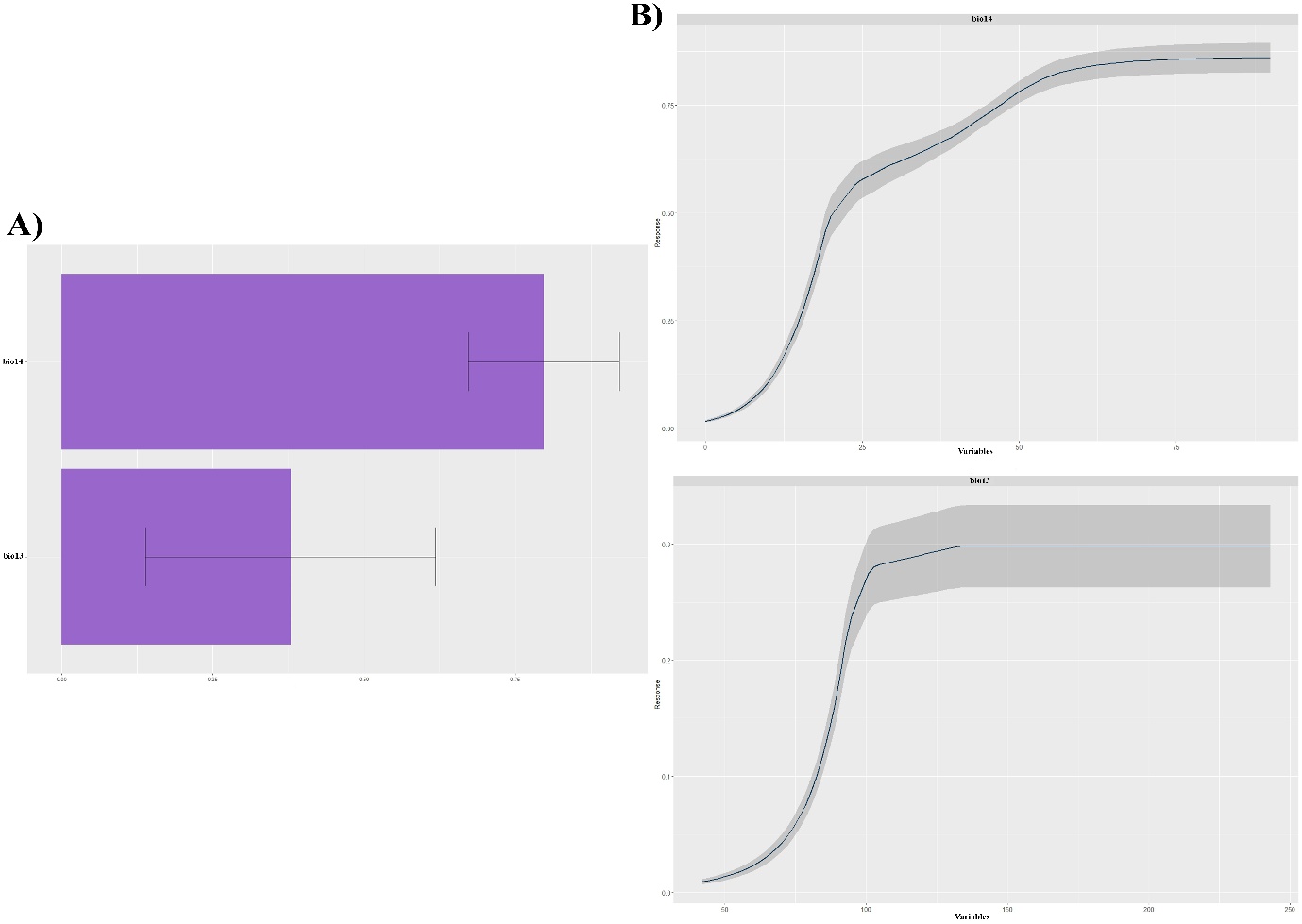


**Figure S7.** A) It shows relative variable importance for *Sphagnum fallax*, B) This represents the response curve of the most important variables to model predicting *Sphagnum fallax*.


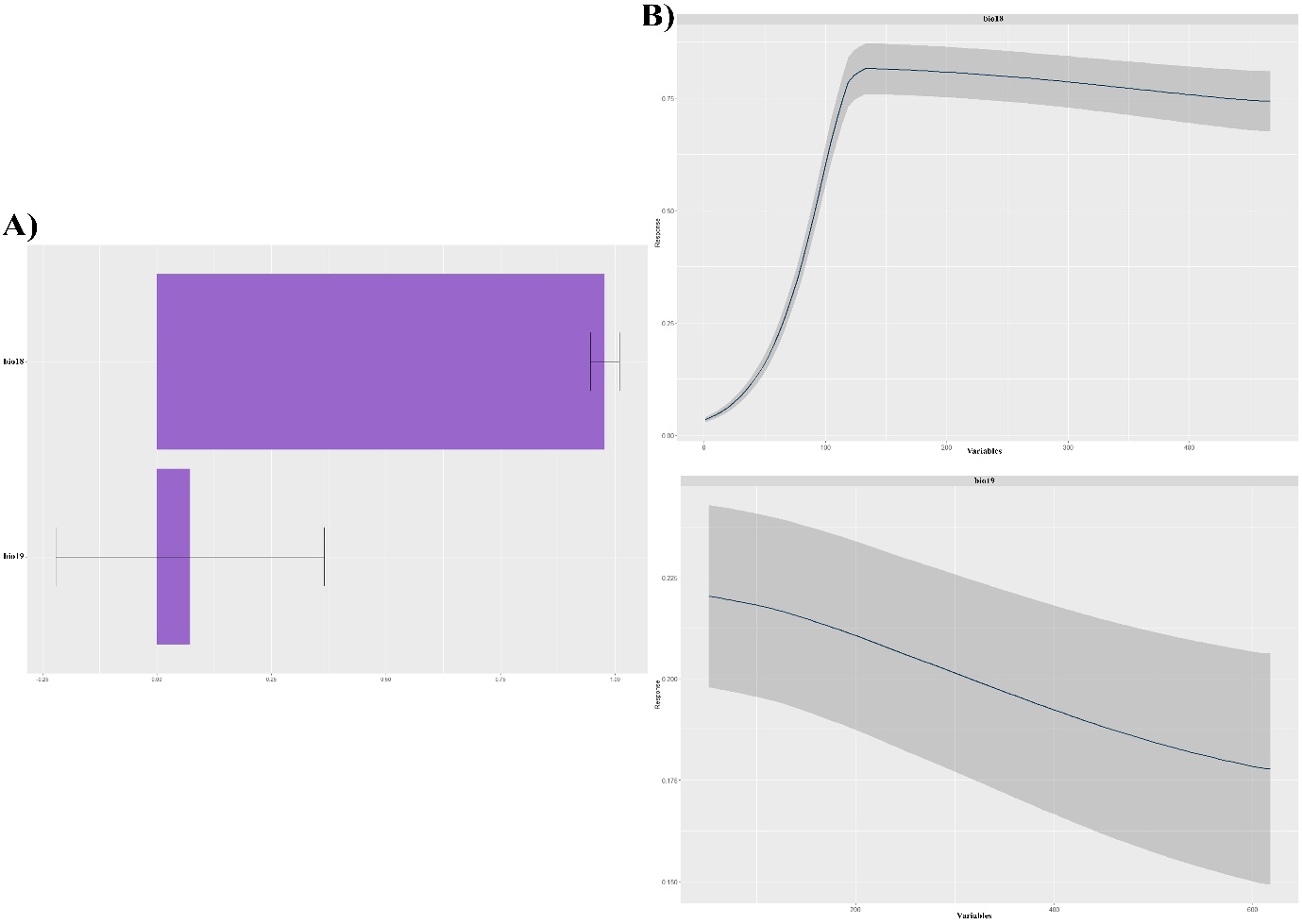


**Figure S8.** A) It shows relative variable importance for *Sphagnum fuscum*, B) This represents the response curve of the most important variables to model predicting *Sphagnum fuscum*.


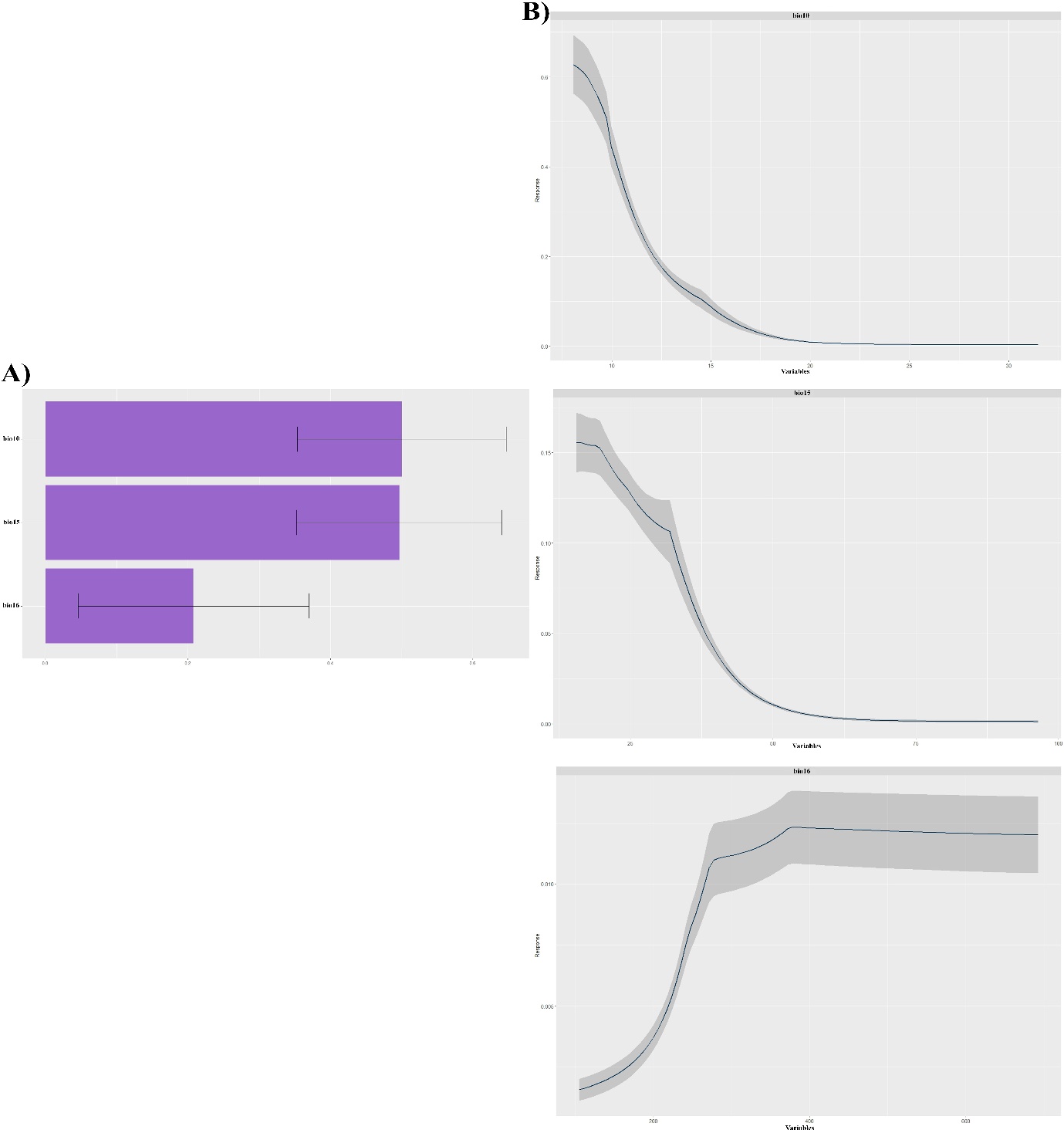


**Figure S9.** A) It shows relative variable importance for *Sphagnum girgensohnii*, B) This represents the response curve of the most important variables to model predicting *Sphagnum girgensohnii*.


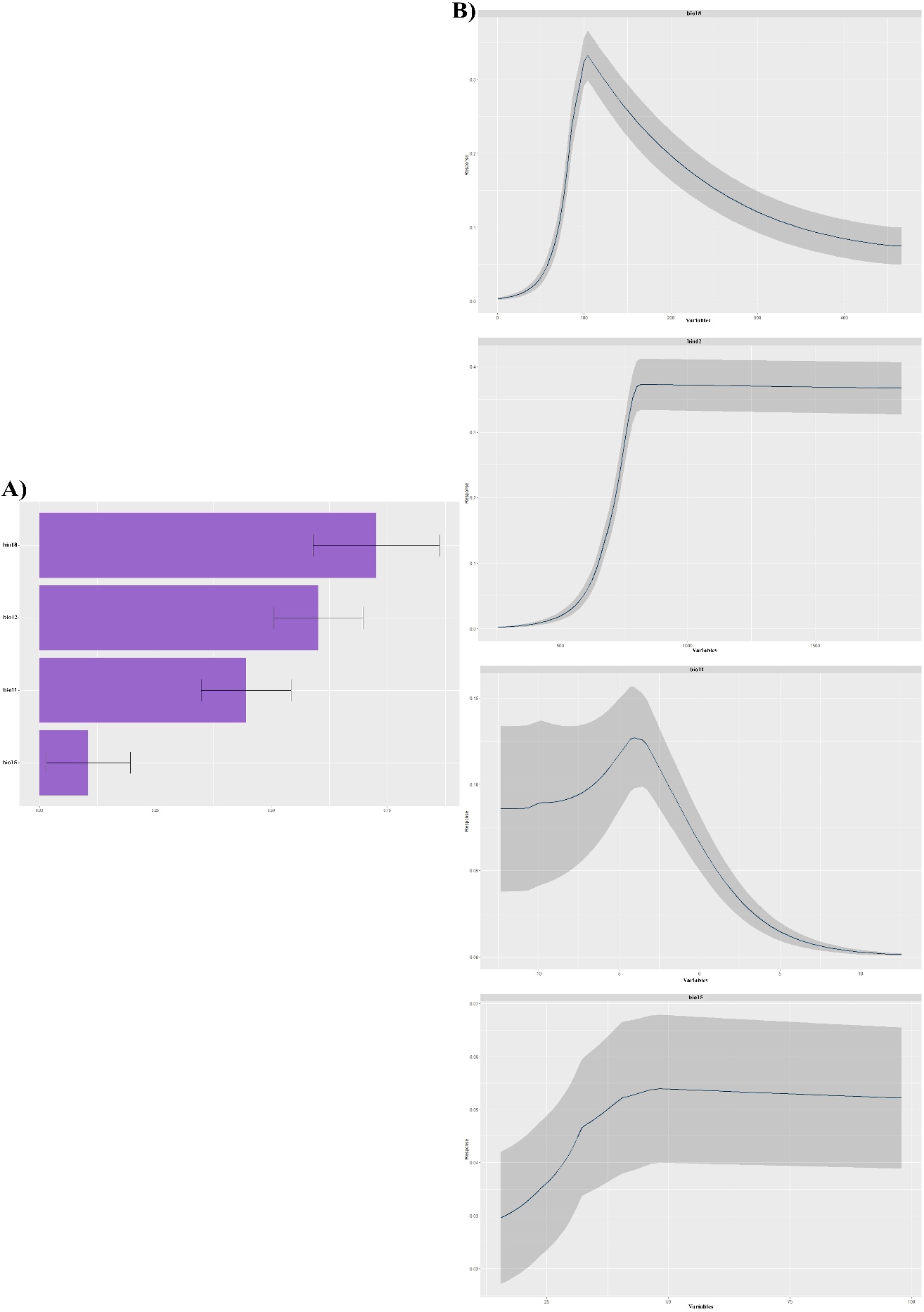


**Figure S10.** A) It shows relative variable importance for *Sphagnum inundatum*, B) This represents the response curve of the most important variables to model predicting *Sphagnum inundatum*.


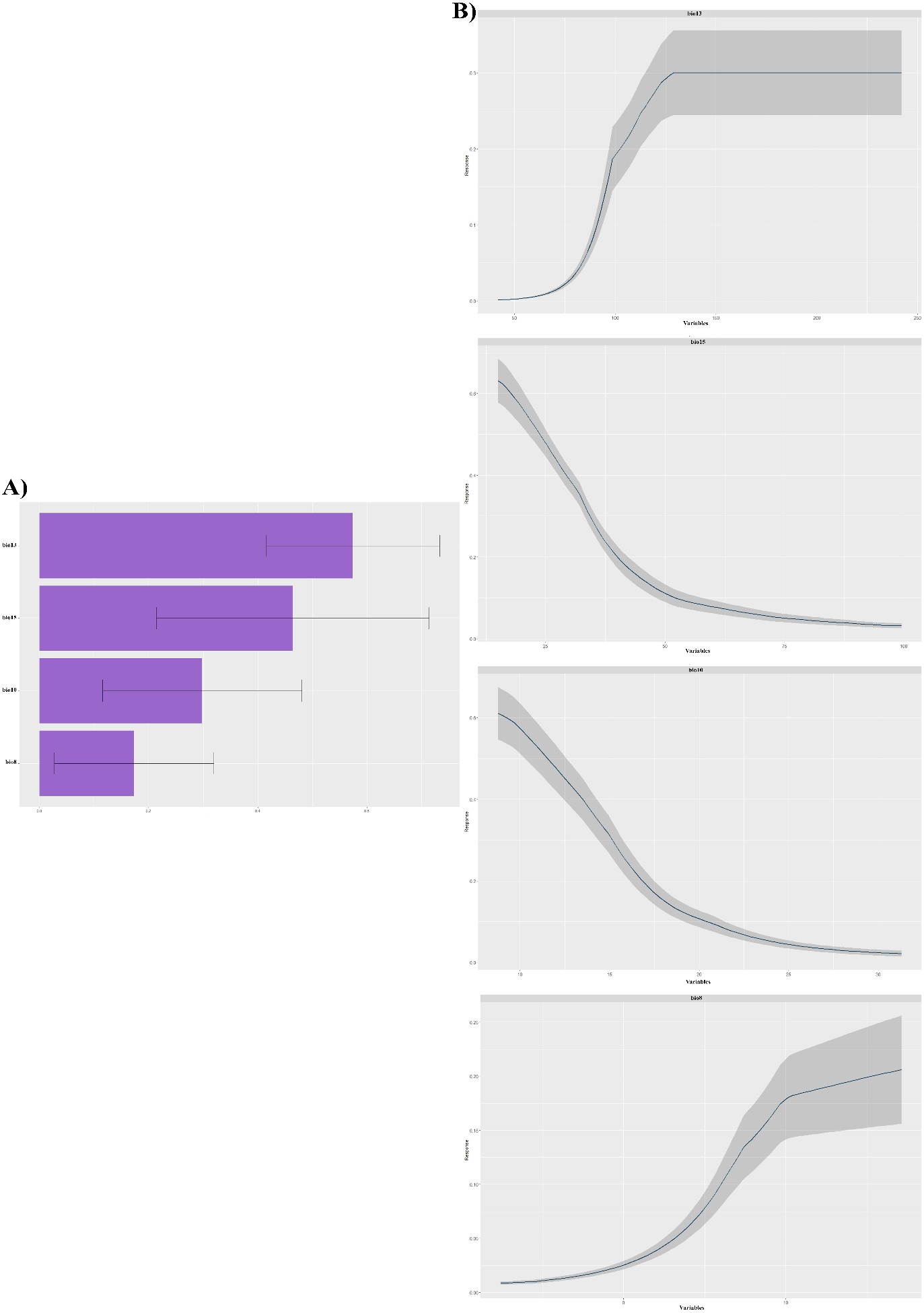


**Figure S11.** A) It shows relative variable importance for *Sphagnum palustre*, B) This represents the response curve of the most important variables to model predicting *Sphagnum palustre*.


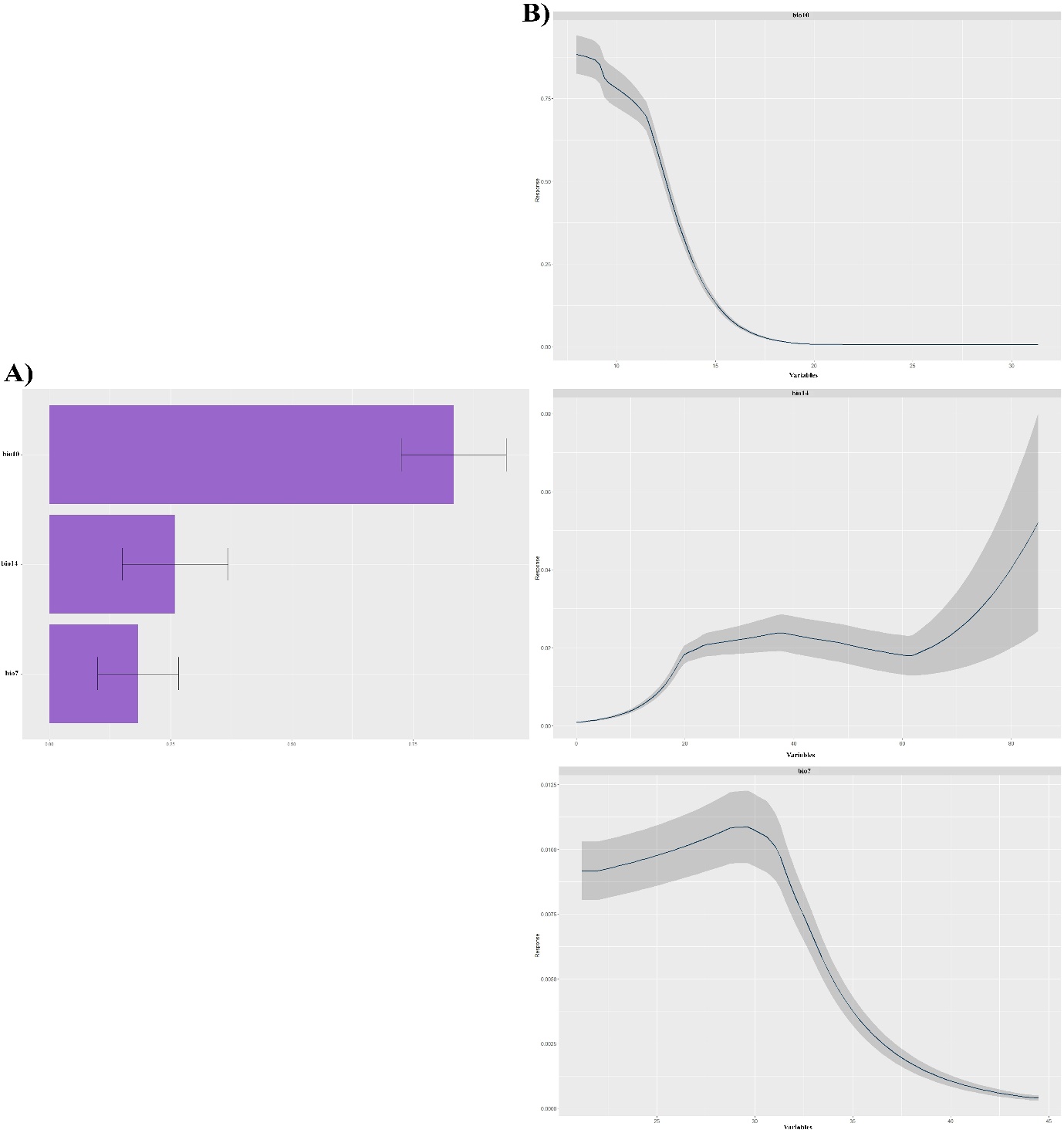


**Figure S12.** A) It shows relative variable importance for *Sphagnum platyphyllum*, B) This represents the response curve of the most important variables to model predicting *Sphagnum platyphyllum*.


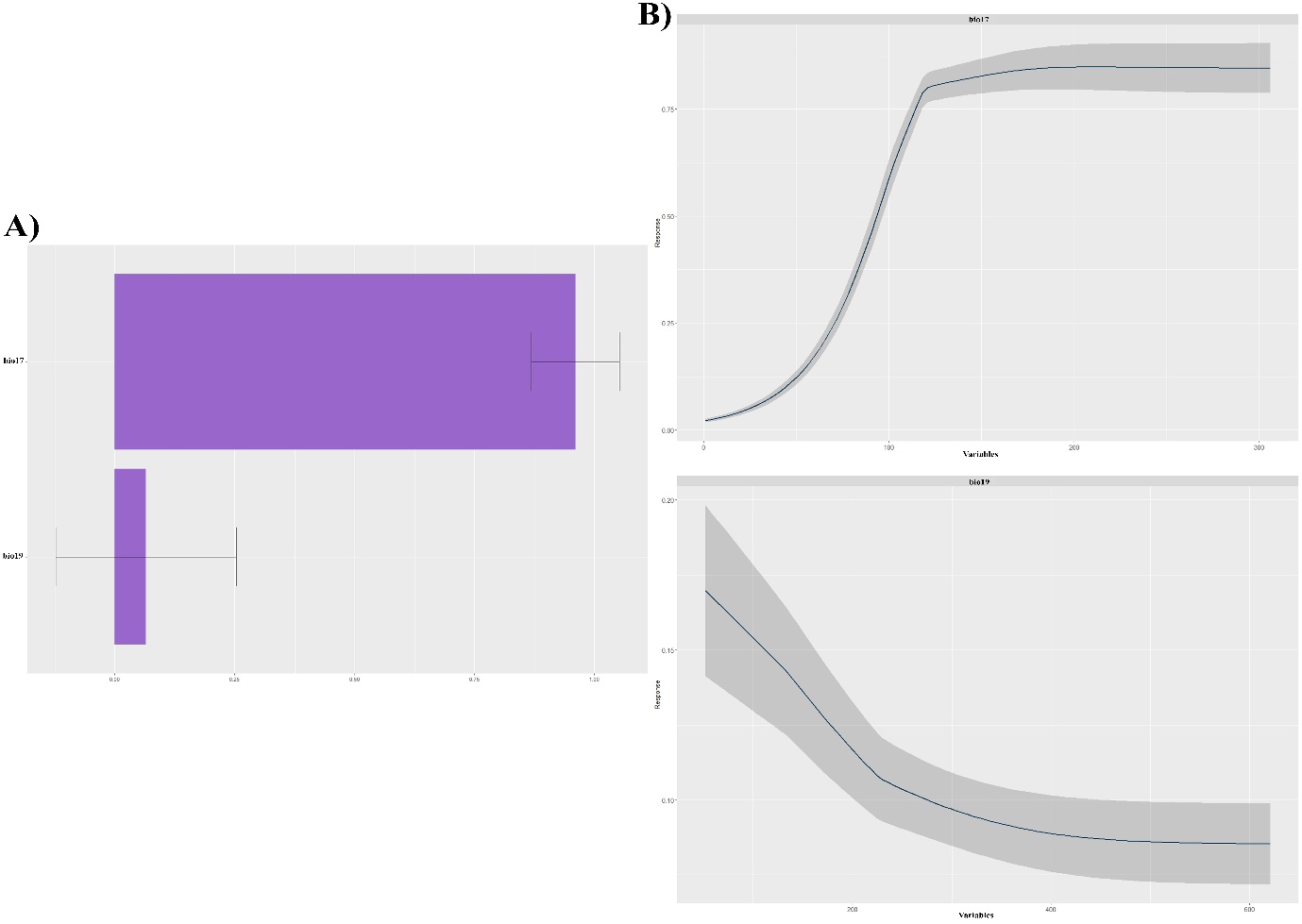


**Figure S13.** A) It shows relative variable importance for *Sphagnum rubellum*, B) This represents the response curve of the most important variables to model predicting *Sphagnum rubellum*.


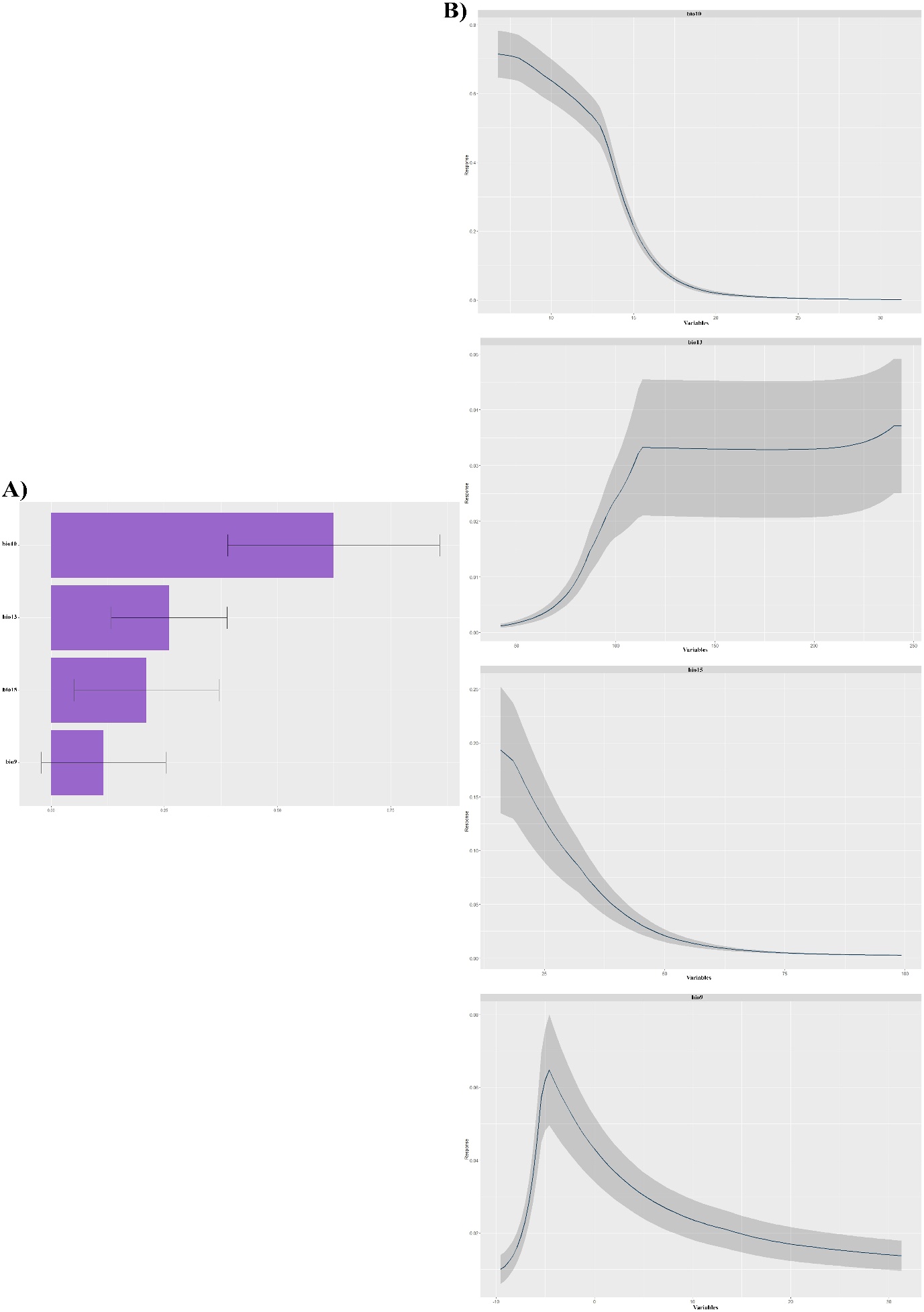


**Figure S14.** A) It shows relative variable importance for *Sphagnum squarrosum*, B) This represents the response curve of the most important variables to model predicting *Sphagnum squarrosum*.


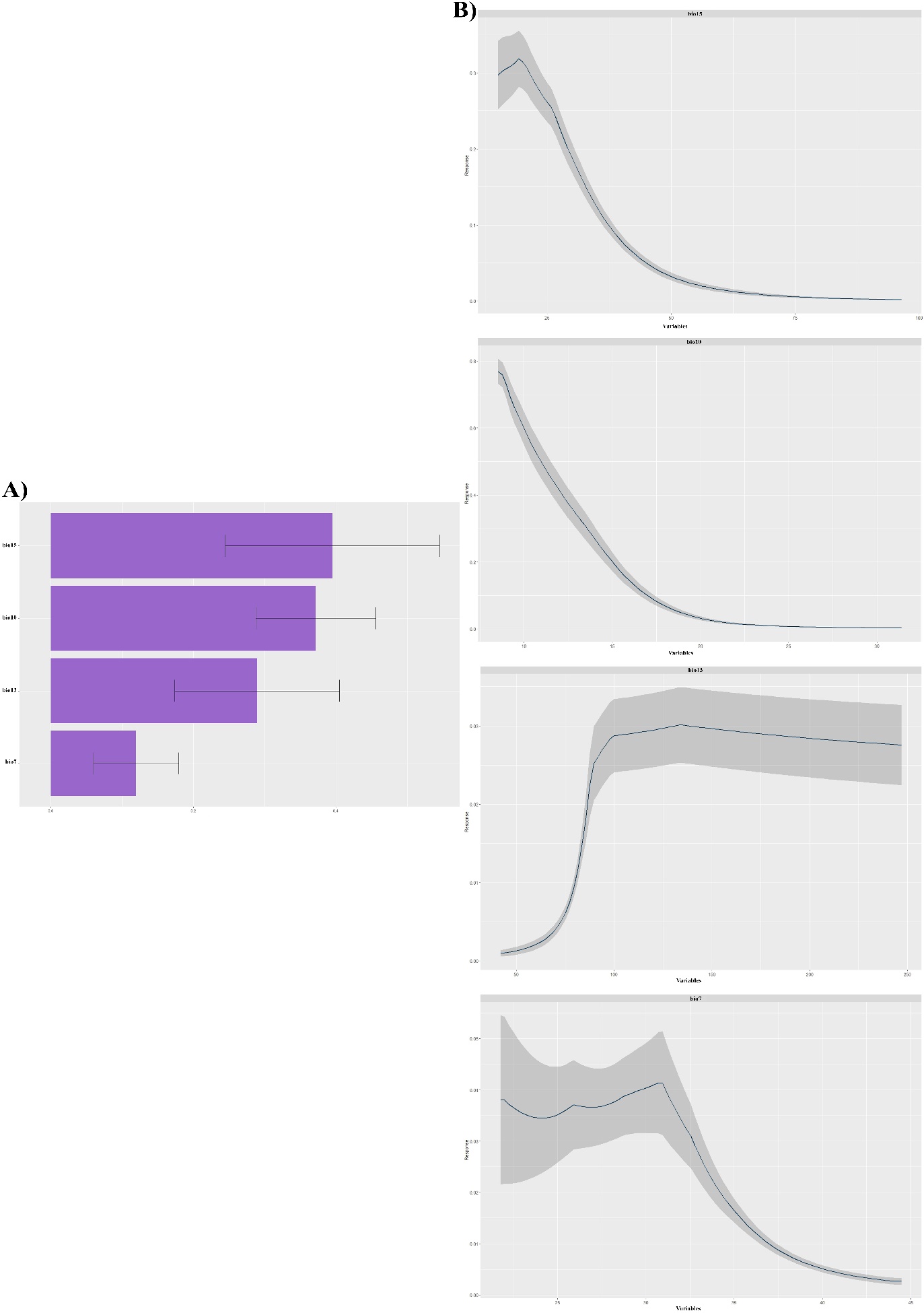


**Figure S15.** A) It shows relative variable importance for *Sphagnum subsecundum*, B) This represents the response curve of the most important variables to model predicting *Sphagnum subsecundum*.


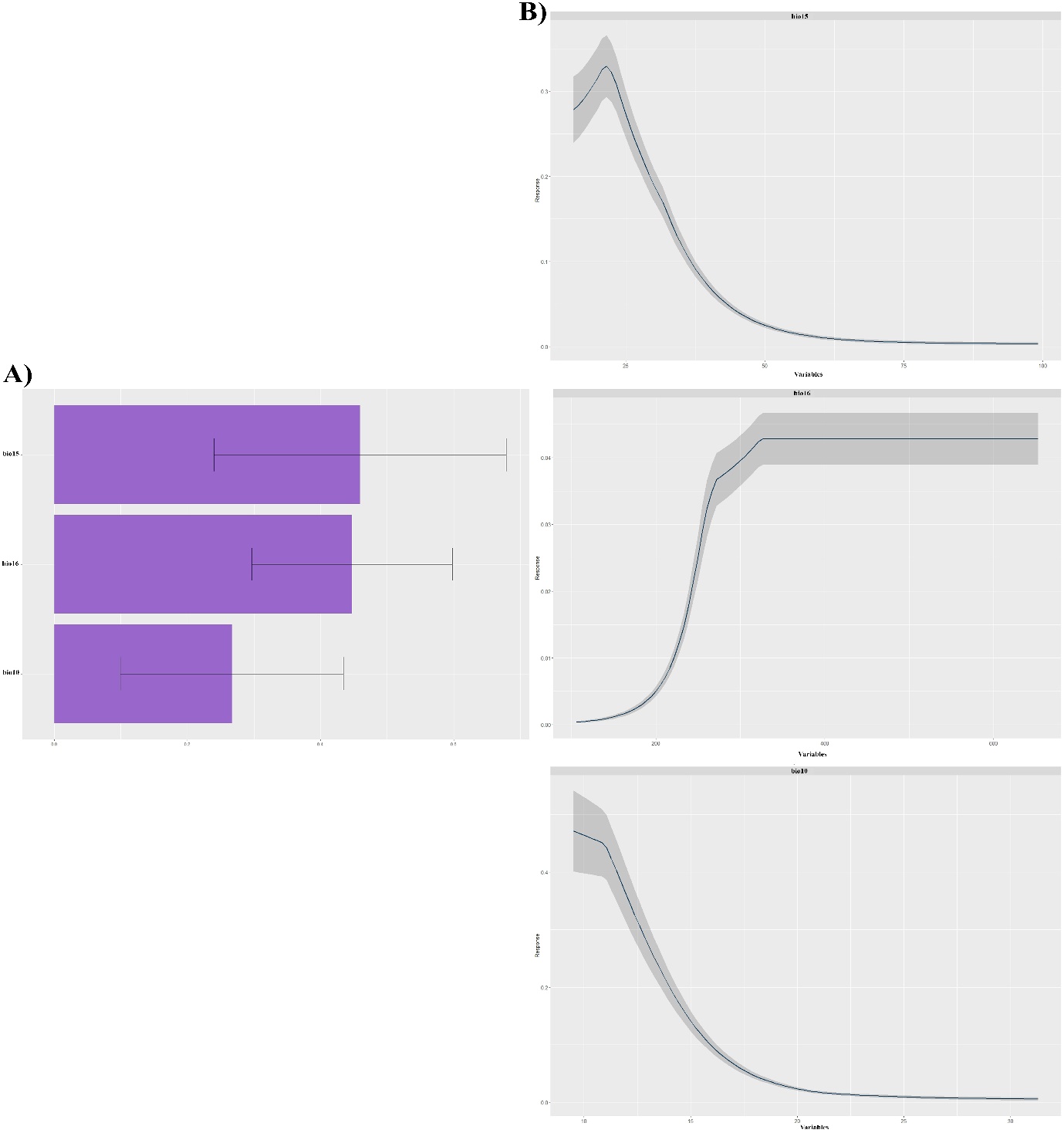


**Figure S16.** A) It shows relative variable importance for *Sphagnum teres*, B) This represents the response curve of the most important variables to model predicting *Sphagnum teres*.


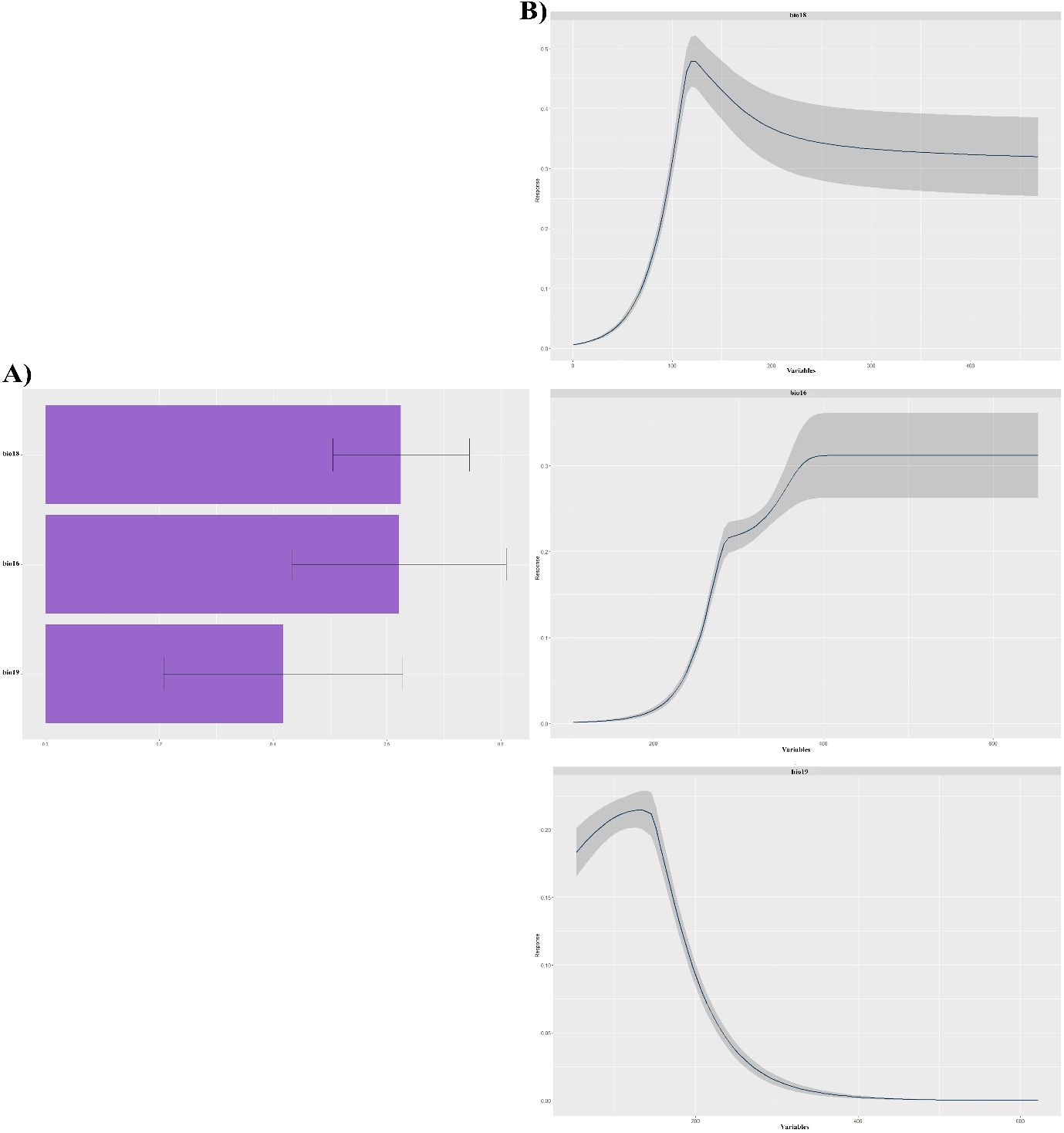


**Figure S17.** A) It shows relative variable importance for *Sphagnum warnstorfii*, B) This represents the response curve of the most important variables to model predicting *Sphagnum warnstorfii*.
